# Medications Used Among Nonhospitalized Pregnant Women With COVID‐19: A Prospective Individual Patient Data Meta‐Analysis in Europe and North America

**DOI:** 10.1002/pds.70303

**Published:** 2025-12-25

**Authors:** Odette de Bruin, Emeline Maisonneuve, Eimir Hurley, Hedvig M. E. Nordeng, Anick Bérard, Odile Sheehy, Padma Kaul, Mayura U. Shinde, Austin Cosgrove, Jennifer G. Lyons, Elizabeth Messenger‐Jones, Maria E. Kempner, Sengwee Toh, Wei Hua, José J. Hernández‐Muñoz, Leyla Sahin, Carolyn E. Cesta, David Hägg, Rosa Gini, Olga Paoletti, Beatriz Poblador‐Plou, Sue Jordan, Daniel Thayer, Clara L. Rodríguez‐Bernal, Francisco Sánchez‐Sáez, Régis Lassalle, Marie‐Agnès Bernard, Ema Alsina, Fariba Ahmadizar, Guillaume Favre, Alice Panchaud, Kitty W. M. Bloemenkamp, Kelly Plueschke, Corinne de Vries, Satu J. Siiskonen, Miriam C. J. M. Sturkenboom, the CONSIGN Collaboration Group, Benjamin P. Geisler, Mark Walker, Steven Hawken, Sasha Bernatsky, Sherif Eltonsy, Emma Hoffman, Andrew B. Petrone, Jolene Mosley, Jenice Ko, Claudia Bartolini, Giuseppe Roberto, Giorgio Limoncella, Anna Girardi, Giulia Hyeraci, Antonio Gimeno‐Miguel, Jonás Carmona‐Pírez, Antonio Poncel‐Falcó, Aida Moreno‐Juste, Alexandra Prados‐Torres, Ian Farr, Saira Ahmed, Ieuan Scanlon, Gabriel Sanfélix‐Gimeno, Isabel Hurtado, Anibal Garcia‐Sempere, Salvador Peiro, Jérémy Jové, Dunia Sakr, Cécile Droz‐Perroteau, David Baum, Hilde M. Engjom, Mònica Sabaté Gallego, Elena Ballarín Alins, Cristina Aguilera Martin, Melissa Kampman, Celline Brasil

**Affiliations:** ^1^ Department of Data Science & Biostatistics, Julius Global Health University Medical Center Utrecht (UMCU) Utrecht the Netherlands; ^2^ Department of Obstetrics, Division of Woman and Baby, Wilhelmina Children's Hospital University Medical Center Utrecht (UMCU) Utrecht the Netherlands; ^3^ Institute of Primary Health Care (BIHAM), University of Bern Bern Switzerland; ^4^ Graduate School for Health Sciences (GHS) University of Bern Bern Switzerland; ^5^ Materno‐Fetal and Obstetrics Research Unit, Woman‐Mother‐Child Department Lausanne University Hospital Lausanne Switzerland; ^6^ Pharmacoepidemiology and Drug Safety Research Group, Department of Pharmacy University of Oslo (UiO) Oslo Norway; ^7^ Department of Child Health and Development Norwegian Institute of Public Health Oslo Norway; ^8^ Faculty of Pharmacy University of Montreal Montreal Quebec Canada; ^9^ Centre Hospitalier Universitaire (CHU) de Sainte‐Justine Montreal Quebec Canada; ^10^ University of Alberta Edmonton Alberta Canada; ^11^ Department of Population Medicine Harvard Medical School and Harvard Pilgrim Health Care Institute Boston Massachusetts USA; ^12^ Department of Population Medicine Harvard Pilgrim Health Care Institute Boston Massachusetts USA; ^13^ Office of Surveillance and Epidemiology Center for Drug Evaluation and Research, U.S. Food and Drug Administration Silver Spring Maryland USA; ^14^ Office of New Drugs, Center for Drug Evaluation and Research U.S. Food and Drug Administration Silver Spring Maryland USA; ^15^ Department of Medicine Solna Centre for Pharmacoepidemiology, Karolinska Institutet Stockholm Sweden; ^16^ Tuscan Regional Healthcare Agency Florence Italy; ^17^ EpiChron Research Group, Aragon Health Sciences Institute (IACS), IIS Aragón Miguel Servet University Hospital Zaragoza Spain; ^18^ Network for Research on Chronicity, Primary Care and Health Promotion (RICAPPS) Research Network on Health Services in Chronic Diseases, Institute of Health Carlos III Madrid Spain; ^19^ Faculty of Medicine, Health and Life Science Swansea University Swansea UK; ^20^ Health Services Research and Pharmacoepidemiology Unit Foundation for the Promotion of Health and Biomedical Research of Valencia Region Valencia Spain; ^21^ Bordeaux PharmacoEpi, INSERM CIC‐P1401 Université de Bordeaux Bordeaux France; ^22^ Service of Pharmacy Lausanne University Hospital and University of Lausanne Lausanne Switzerland; ^23^ European Medicines Agency Amsterdam the Netherlands; ^24^ Division of Pharmacoepidemiology and Clinical Pharmacology Utrecht Institute for Pharmaceutical Sciences (UIPS), Utrecht University Utrecht the Netherlands

**Keywords:** CONSIGN, COVID‐19, medication use, meta‐analysis, pregnancy

## Abstract

**Aim:**

To estimate the prevalence of medication use in nonhospitalized pregnant women with COVID‐19.

**Methods:**

A prospective two‐stage individual patient meta‐analysis across 10 data sources in Europe and North America studied medication use among nonhospitalized pregnant women with COVID‐19 between January 2020 and December 2022. Comparisons were made between medication use within 30 days pre‐ and post‐COVID‐19 diagnosis in this cohort and two comparator groups: pregnant women without COVID‐19 and nonpregnant women with COVID‐19. Prevalence estimates were pooled using a random‐effects model stratified by trimester.

**Results:**

50 335 nonhospitalized pregnant women with COVID‐19 were identified. The pooled prevalence of antibacterial use in the third trimester was higher post‐COVID‐19 diagnosis (6.8%, 95% confidence interval [CI] = 5.5–8.4, *I* ^2^ = 94%) compared with the same women pre‐COVID‐19 (3.9%, 95% CI = 3.1–4.9, *I* ^2^ = 89%). Overall, pregnant women with COVID‐19 had higher medication use compared to pregnant women without COVID‐19, although the CIs of the prevalence overlapped. Post‐COVID‐19, antithrombotic prevalence was 4.5% (95% CI = 1.1–16.5, *I* ^2^ = 100%) among pregnant women with COVID‐19 in the third trimester, compared to 2.1% (95% CI = 1.2–3.6, *I* ^2^ = 99%) among those without COVID‐19 in the third trimester. Compared to nonpregnant women with COVID‐19, pregnant women with COVID‐19 were less likely to be prescribed analgesics, antiprotozoals, corticosteroids, psychoanaleptics and psycholeptics, and more likely to be prescribed antithrombotics, cough and cold and nasal preparations, and drugs used in diabetes across all trimesters. High heterogeneity existed in nearly all analyses.

**Conclusion:**

This international meta‐analysis reveals low medication use and country‐specific variations, enhancing insight into the management of COVID‐19 in nonhospitalized pregnant women. Higher antithrombotic use post‐COVID‐19 suggests prophylactic treatment in this population, but variation between countries emphasizes the challenges of combining multinational data.

## Summary

- This meta‐analysis, combining data from 50 335 nonhospitalized pregnant women with COVID‐19 across seven countries, compared the prevalence of medication use 30 days before and after COVID‐19 diagnosis in this cohort, alongside two comparator groups: pregnant women without COVID‐19 and nonpregnant women with COVID‐19.
- Overall, medication use in nonhospitalized pregnant women with COVID‐19 was low and varied widely across data sources, with antibacterials, antithrombotic agents, and analgesics being the most frequently prescribed medications.
- Nonhospitalized pregnant women with COVID‐19 were more likely to receive antithrombotic agents compared to both pregnant women without COVID‐19 and nonpregnant women with COVID‐19, reflecting global thrombosis prophylaxis guidelines for pregnant individuals with COVID‐19.
- Nonhospitalized pregnant women with COVID‐19 were prescribed certain medications less frequently than nonpregnant women with COVID‐19, suggesting potential undertreatment due to fetal safety concerns.
- The findings underscore the importance of international collaboration and standardized data collection to improve surveillance and support evidence‐based decision‐making in pregnancy pharmacotherapy, particularly during public health emergencies.

## Plain Language Summary

This international study examined the use of medications in 50 335 pregnant women with COVID‐19 who were not hospitalized, across seven countries, from January 2020 to December 2022. Researchers compared medication use before and after the COVID‐19 diagnosis in these women, as well as with two other groups: pregnant women without COVID‐19 and nonpregnant women with COVID‐19. The findings revealed that pregnant women with COVID‐19 generally used more medications than those without COVID‐19, though the difference was not significant. Specifically, they were more likely to be prescribed blood thinners after their diagnosis, particularly in the third trimester. Pregnant women with COVID‐19 were also less likely to receive painkillers, anti‐parasitic drugs, steroids, or medications for anxiety compared to nonpregnant women with COVID‐19. Medication use varied by country, with blood thinners commonly used as a preventive measure against blood clots. These results provide important insights into how COVID‐19 was managed in nonhospitalized pregnant women and can help guide evidence‐based decision‐making in different healthcare settings. The study also highlights the challenges of combining data from multiple countries, as differences in healthcare systems, data collection methods, and study periods contributed to variation in the results.

## 1 Introduction

The COVID‐19 pandemic has raised significant concerns for pregnant women, their healthcare providers, governmental authorities, and medicines regulators. Pregnant women were notably absent from most pivotal clinical trials assessing the effectiveness and safety of medications for treating and preventing COVID‐19 [1, 2]. Consequently, substantial knowledge gaps remain regarding the effects of these treatments across all trimesters of pregnancy and their impact on maternal and perinatal outcomes. Moreover, little is known about the specific medications used by pregnant women with COVID‐19 in real‐world settings.

Although some studies have explored medication use in pregnant women with COVID‐19, most have focused on hospital‐based care. A systematic review from October 2021, including six studies involving 599 pregnant women, examined the inpatient use of antivirals, systemic corticosteroids, antibiotics, and immunotherapy but excluded anticoagulants. The small sample size limited the ability to conduct a meta‐analysis and draw firm conclusions [3]. Another meta‐analysis assessed medication use among 1742 pregnant patients, but only two studies with very few cases provided stratified results by disease severity [4]. To the best of our knowledge, no published meta‐analysis has addressed medication use among nonhospitalized pregnant women with COVID‐19.

To address this evidence gap, our international collaborative effort, the COVID‐19 infectiOn aNd medicineS In preGnancy (CONSIGN) project was conducted within the EU PE&PV (Pharmacoepidemiology and Pharmacovigilance) Research Network and funded by the European Medicines Agency (EMA). The project aimed to better characterize medication use among pregnant women with COVID‐19 by leveraging both evidence from the secondary use of data provided by the IMI‐funded ConcePTION tools and network, referred to as CONSIGN electronic health records (EHRs) study, as well as through primary data collections from the COVI‐PREG and International Network of Obstetric Survey Systems (INOSS) [5, 6, 7, 8, 9, 10]. The findings indicated that the utilization of medications to treat COVID‐19 during pregnancy is rare, strongly associated with the severity of the disease, and subject to change over time since the onset of the pandemic [5, 6, 7, 8, 9, 10, 11]. However, due to the limited and varying number of pregnant women receiving treatment for COVID‐19, drawing definitive conclusions remained highly challenging. Following discussions with the International Coalition for Medicines Regulatory Agencies (ICMRA) and a landscape analysis of ongoing studies, a prospective two‐stage individual patient data (IPD) meta‐analysis was conducted within the CONSIGN project, incorporating all identified secondary data sources [12, 13, 14]. The focus of this meta‐analysis was on outpatient medication usage, aiming to describe the utilization of medications for treating COVID‐19 among nonhospitalized pregnant women across trimesters of pregnancy.

## 2 Methods

We conducted a prospective two‐stage IPD meta‐analysis, pooling the results from analyses conducted in different data sources utilizing secondary administrative and EHR data, or medical claims data containing information on pregnancies affected by COVID‐19 between January 2020 and December 2022 [15, 16]. The study is registered in the HMA‐EMA catalog, or real‐world data studies with the identifier EUPAS40317, and the protocol and statistical analysis plan (SAP) are available online [14]. To the extent possible, we adhered to the PRISMA‐IPD and Cochrane guidelines for prospective meta‐analysis [17, 18].

### 2.1 Data Source Selection Process

The EMA and CONSIGN leadership engaged through ICMRA with the U.S. Food and Drug Administration (FDA) and Health Canada to foster international collaboration on using medications and their effects on managing COVID‐19 during pregnancy. Identified initiatives were requested to share their analysis plans and additional materials, and meetings were scheduled to discuss the eligibility of participation in the meta‐analysis. To be eligible, networks or individual data sources needed access to population‐based healthcare databases capable of identifying the start and end of pregnancies and linking them to COVID‐19 diagnosis and medication records. Furthermore, they needed to implement fully or partially the CONSIGN EHR study protocol (EUPAS39438) [6, 10, 12, 13].

**FIGURE 1:**
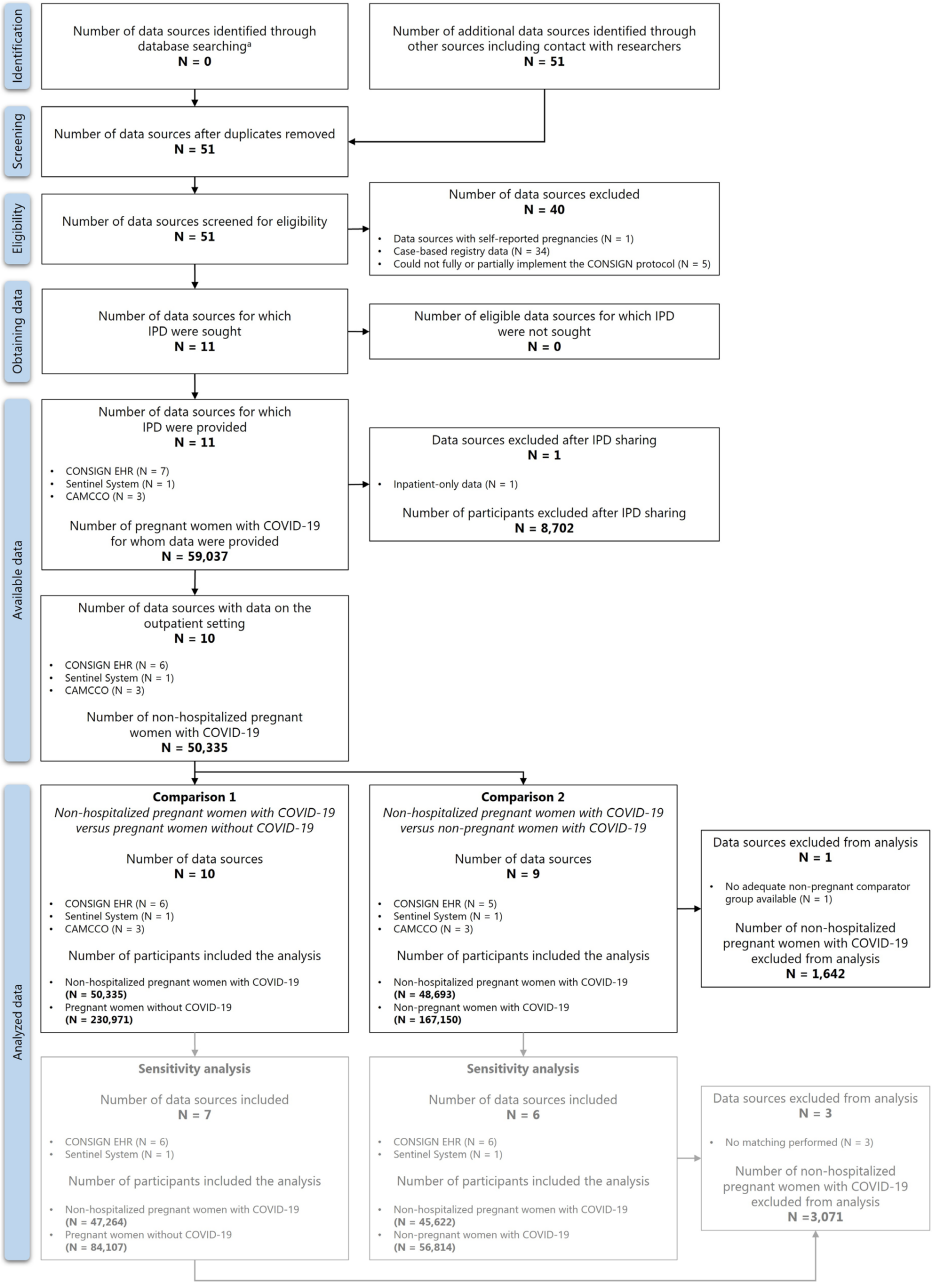
Flowchart of the selection process of networks and data sources, and the number of participants included in the meta‐analysis using secondary healthcare data.

Fifty‐one networks and data sources reporting medication use in pregnant women with COVID‐19 were identified. Of these, 16 were based on the secondary use of data, but 5 were excluded as they could not implement the CONSIGN protocol. Ultimately, individual‐level data were obtained from 11 data sources across three research initiatives (Figure 1). The characteristics of these data sources are presented in Table S2.

The CONSIGN EHR study provided data from seven electronic healthcare registries in six European countries (Tuscany, Italy; France; Valencia, Spain; Aragon, Spain; Wales, UK; Norway; and Sweden). The data sources included general practice (primary care) databases and record linkage of demographic data, registers covering primary and secondary care, and prescribing or dispensing registers (Table S2). The data were analyzed in a distributed manner using a common data model (CDM) and common analytics developed by the IMI‐funded ConcePTION project [5, 6, 10].

The Canadian Mother–Child Cohort (CAMCCO) Active Surveillance Initiative included data from three provinces, Alberta, Manitoba, and Ontario, and their results are presented separately. Data came from medical services, prescription drugs, hospitalization archives, and COVID‐19 testing program results (Table S2). CAMCCO has developed standardized and harmonized tools based on the Quebec Pregnancy/Child Cohort (QPC) [19, 20].

The U.S. FDA Sentinel System included the most recently refreshed data from seven Data Partners (DP) participating in the Rapid COVID‐19 Sentinel Distributed Database, including four national claims‐insurers and three DP with regional integrated care delivery systems (Table S2). Sentinel wrote its own study protocol focusing on COVID‐19 in pregnancy and implemented the key aims and methods of the CONSIGN EHR study protocol [21].

### 2.2 Study Population and Data Items

To standardize data semantics, we reviewed protocols, codebooks, and definitions of populations, exposures, outcomes, and covariates across data sources. The study population comprised an index cohort of pregnant women with a recorded COVID‐19 diagnosis during their pregnancies between January 2020 and the latest available data from each data source (Table S2). Two external comparator cohorts were used: (i) pregnant women without COVID‐19 and (ii) nonpregnant women of childbearing age with COVID‐19. In CONSIGN EHR and the Sentinel System, the external comparator cohorts were matched to the index cohort using pregnancy trimester (for the pregnant comparator), calendar month of COVID‐19 diagnosis (for the nonpregnant comparator), and maternal age group. For the pregnant comparators, uninfected pregnant women were assigned the same index date as the COVID‐19 diagnosis date of their matched counterpart. Likewise, nonpregnant comparators were assigned the same pregnancy trimester as their matched pregnant counterparts. CAMCCO did not deploy matching. Following matching, analyses were restricted to nonhospitalized pregnant women with COVID‐19, defined as those with a recorded positive test or diagnosis and no subsequent hospital admission with a primary or secondary diagnosis of COVID‐19 within a 4‐week period (Figure 1). Alternatively, in settings where the reason for hospital admission was available, women admitted due to COVID‐19 were excluded from the nonhospitalized cohort.

Data source‐specific information to determine pregnancy start and end dates and COVID‐19 diagnosis is provided in Table S3. Across all sites, the start of a pregnancy was defined as the estimated first day of the last menstrual period (LMP), and the end of a pregnancy was defined as the date of birth (or non‐live births in sites collecting this information). The Sentinel System algorithm used to detect pregnancies was based solely on the identification of live births. The definitions of trimesters of pregnancy are slightly different among the three included networks (Table S4). COVID‐19 cases were identified through records of COVID‐19 in surveillance systems, diagnostic codes in healthcare records, and/or laboratory results (Table S3). In all data sources, the identification of confirmed COVID‐19 diagnoses was possible through the use of polymerase chain reaction (PCR) or antigen tests.

Medication groups considered of special relevance to COVID‐19 were identified from the World Health Organization (WHO) and National Institutes of Health (NIH) guidelines and included analgesics, anthelminthics, anti‐inflammatory and antirheumatic products, antibacterials for systemic use, antigout preparations, antihypertensives, antimycobacterials, antimycotics for systemic use, antineoplastic agents, antiprotozoals, antithrombotic agents, antivirals for systemic use, corticosteroids for systemic use, cough and cold preparations, drugs for obstructive airway diseases, drugs used in diabetes, immune sera and immunoglobulins, immunostimulants, immunosuppressants, nasal preparations, psychoanaleptics, and psycholeptics [22, 23]. The one‐month prevalence of each medication was assessed using the Anatomical Therapeutic Classification (ATC) at level 2 (Table S5). The numerator captured whether the medication was prescribed or dispensed within a 30‐day risk window, and the denominator represented the number of women in the 30 days prior to the date of COVID‐19 diagnosis, and separately in the 30 days following diagnosis. For pregnant comparators without COVID‐19, in data sources where matching was performed, the index date of their matched COVID‐19 case was used to define the same 30‐day exposure windows. In data sources without matching, pregnant women without COVID‐19 infection were considered exposed to a given trimester if they filled at least one prescription during that trimester.

Covariates of interest included: maternal age, trimester of pregnancy, at‐risk medical conditions for severe COVID‐19, risk conditions for obstetric complications, and calendar month of COVID‐19 diagnosis (Table S4).

### 2.3 Statistical Analysis

The protocol and SAP are publicly available elsewhere [14]. A common R‐script based on the ConcePTION CDM structure was created for the data sources participating in the CONSIGN EHR study, except at Karolinska Institutet in Sweden, where they programmed their own analysis in SAS according to the SAP. CAMCCO and Sentinel used the CONSIGN EHR study SAP and ran their own scripts in SAS. All data sources delivered aggregated results, including counts, proportions, and 95% confidence intervals (CIs), in prespecified shell table formats.

The statistical analyses were undertaken with R software, version 4.2.2 [24]. A meta‐analysis of proportions was conducted using the “metaprop” function, enabling the calculation of a combined effect estimate with corresponding 95% CI. The Generalized Linear Mixed Model (GLMM) method assumes a random effects model and was employed to account for potential heterogeneity among individual study estimates [25]. Specifically, the PLOGIT function was used to estimate the logit‐transformed proportions. The use of the PLOGIT function allows for a more accurate estimation of proportion, especially when dealing with small sample sizes and sparse data [26, 27].

Comparisons were made between the prevalence of medication use within 30 days pre‐ and 30 days post‐COVID‐19 diagnosis in pregnant women with COVID‐19 and in two comparator groups: pregnant women without COVID‐19 and nonpregnant women with COVID‐19. Analyses were stratified by pregnancy trimester when the COVID‐19 infection occurred and were limited to nonhospitalized COVID‐19 cases to avoid misclassification of exposure during hospitalization. Forest plots were used to show individual site estimates (with 95% CI) and a diamond to represent the pooled estimate (95% CI) for each outcome of interest. Statistical significance was assessed by examining the overlap of CIs rather than by applying a statistical test. We evaluated heterogeneity with the *I* ^2^ statistic.

Some sites were not allowed to share nonzero event counts < 10, < 6, or < 5. In those cases, we imputed one event to calculate the prevalence. Otherwise, all sites with < 10 events would have been excluded from the analyses, which would have introduced a bias. Where zero events were observed at a site, the model included the prevalence of 0%. Additionally, we performed sensitivity analyses excluding CAMCCO sites due to their lack of matching.

## 3 Results

The study identified 11 data sources in eight countries, providing IPD for a total of 59 037 pregnant women diagnosed with COVID‐19 during pregnancy. Most countries contributed pregnancy data from 2020 to 2021, while the United States provided data through the end of 2022 (Table S2). Among the 59 037 pregnant women with COVID‐19, 50 335 (85.3%) were not hospitalized. As France only had access to inpatient data, it was excluded from the analyses (Figure 1). More than half (50.6%) of the nonhospitalized COVID‐19 infections (50.6%) occurred during the third trimester of pregnancy. The distribution of infections across trimesters varied notably between regions. In particular, Sweden and Manitoba, Canada, had fewer first‐trimester infections compared to other regions (Table 1).

**TABLE 1:** Description of the different pregnancy cohorts positive for COVID‐19.

	Total number of pregnant women with COVID‐19	Total number of nonhospitalized pregnant women with COVID‐19	Nonhospitalized pregnant women with COVID‐19 Trimester 1	Nonhospitalized pregnant women with COVID‐19 in Trimester 2	Nonhospitalized pregnant women with COVID‐19 in Trimester 3
Tuscany, Italy	995	739	181 (24.5%)	261 (35.3%)	297 (40.2%)
France^a^	1069	0	0 (0%)	0 (0%)	0 (0%)
Valencia, Spain	3654	3231	1241 (38.4%)	1032 (31.9%)	958 (29.7%)
Aragon, Spain	951	754	201 (26.7%)	272 (36.1%)	281 (37.3%)
Wales, UK	1941	1642	555 (33.8%)	599 (36.5%)	488 (29.7%)
Norway	1146	879	217 (24.7%)	361 (41.1%)	301 (34.2%)
Sweden	5030	4199	259 (6.2%)	1455 (34.7%)	2485 (59.2%)
Alberta, Canada	2296	1968	571 (29.0%)	706 (35.9%)	691 (35.1%)
Manitoba, Canada	235	170	19 (11.2%)	62 (36.5%)	89 (52.4%)
Ontario, Canada	933	933	270 (28.9%)	303 (32.5%)	360 (38.6%)
US	40 787	35 820	6726 (18.8%)	9562 (26.7%)	19 532 (54.5%)
Total	59 037	50 335	10 240 (20.3%)	14 613 (29.0%)	25 482 (50.6%)

^a^ COVID‐19 cases in France were identified through hospital admissions records with a COVID‐19 diagnosis. Consequently, pregnant women not requiring hospitalization are not included.

### 3.1 Pre‐ and Post‐COVID‐19 Diagnosis Medication Use in Pregnant Women With COVID‐19

**FIGURE 2:**
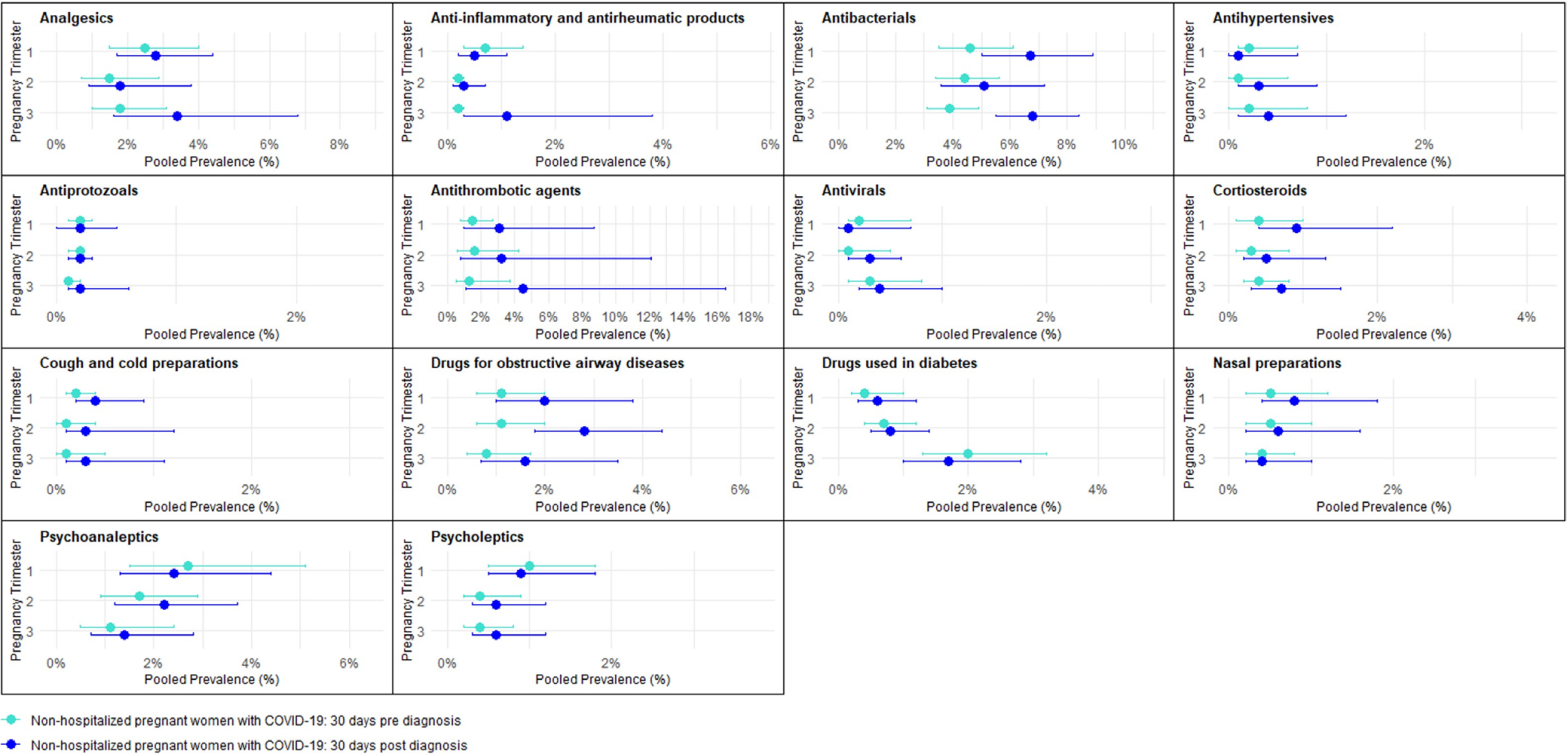
Pooled prevalence of medication use in the 30 days pre‐ and post‐COVID‐19 diagnosis in nonhospitalized pregnant women with COVID‐19 by medication group and pregnancy trimester.

Figure 2 displays the pooled prevalence of medication use in the 30 days pre‐ and post‐COVID‐19 diagnosis in nonhospitalized pregnant women with COVID‐19 by medication group and pregnancy trimester of infection. All medication groups exhibited a low monthly prevalence across the different pregnancy trimesters, with values consistently below 7%. Notably, the pooled prevalence pre‐ and post‐COVID‐19 for anthelminthics, antigout preparations, antimycobacterials, antimycotics, antineoplastic agents, immune sera and immunoglobulins, immunostimulants, and immunosuppressants was found to be < 0.2% across all trimesters. Due to this extremely low prevalence, these medication groups are not depicted in Figure 2. The forest plots showing the pooled prevalence of all medication groups are available in Figures S1–S22. For nearly all medication groups and trimesters, the heterogeneity of the pooled prevalence pre‐ and post‐COVID‐19 diagnosis was high.

Comparisons between the 30 days pre‐ and post‐COVID‐19 diagnosis periods showed a significant increase in the pooled prevalence of the use of antibacterials after COVID‐19 diagnosis in the third pregnancy trimester (pre‐COVID‐19 3.9%, 95% CI = 3.1–4.9, *I* ^2^ = 89% vs. post‐COVID‐19 6.8%, 95% CI = 5.5–8.4, *I* ^2^ = 94%). This increase in antibacterials in the month following COVID‐19 diagnosis was also seen in the first and second pregnancy trimesters, although CIs overlapped (Figure S4). With other medications, including analgesics, antithrombotic agents, corticosteroids, cough and cold preparations, and drugs for obstructive airway diseases, we also observed an increase in the pooled prevalence across all trimesters, but the CIs of the prevalence pre‐ and post‐COVID‐19 diagnosis overlapped (Figure 2). The increase in antithrombotic agents following a COVID‐19 diagnosis was most prominent in the third trimester with a pooled prevalence of 1.3% (95% CI = 0.5–3.7, *I* ^2^ = 97%) pre‐COVID‐19 and 4.5% (95% CI = 1.1–16.5, *I* ^2^ = 100%) post‐COVID‐19, mostly due to the increase in dispensing in European sites (Figure S11).

Additionally, a nonstatistically significant increase was observed for anti‐inflammatory and antirheumatic products, antihypertensives, antivirals, psychoanaleptics, and psycholeptics in the second and third pregnancy trimesters, and for drugs used in diabetes and nasal preparations in the first and second pregnancy trimesters (Figure 2). The increase in anti‐inflammatory and antirheumatic products prescription following a COVID‐19 diagnosis was most prominent in the third pregnancy trimester (pre‐COVID‐19 = 0.2%, 95% CI = 0.1–0.3, *I* ^2^ = 8% vs. post‐COVID‐19 = 1.1%, 95% CI = 0.3–3.8, *I* ^2^ = 98%), mainly attributed to an increase in prevalence in the United States, Manitoba, Canada, and Spain (Figure S3).

### 3.2 Medication Use in Pregnant Women With COVID‐19 and Pregnant Women Without COVID‐19

**FIGURE 3:**
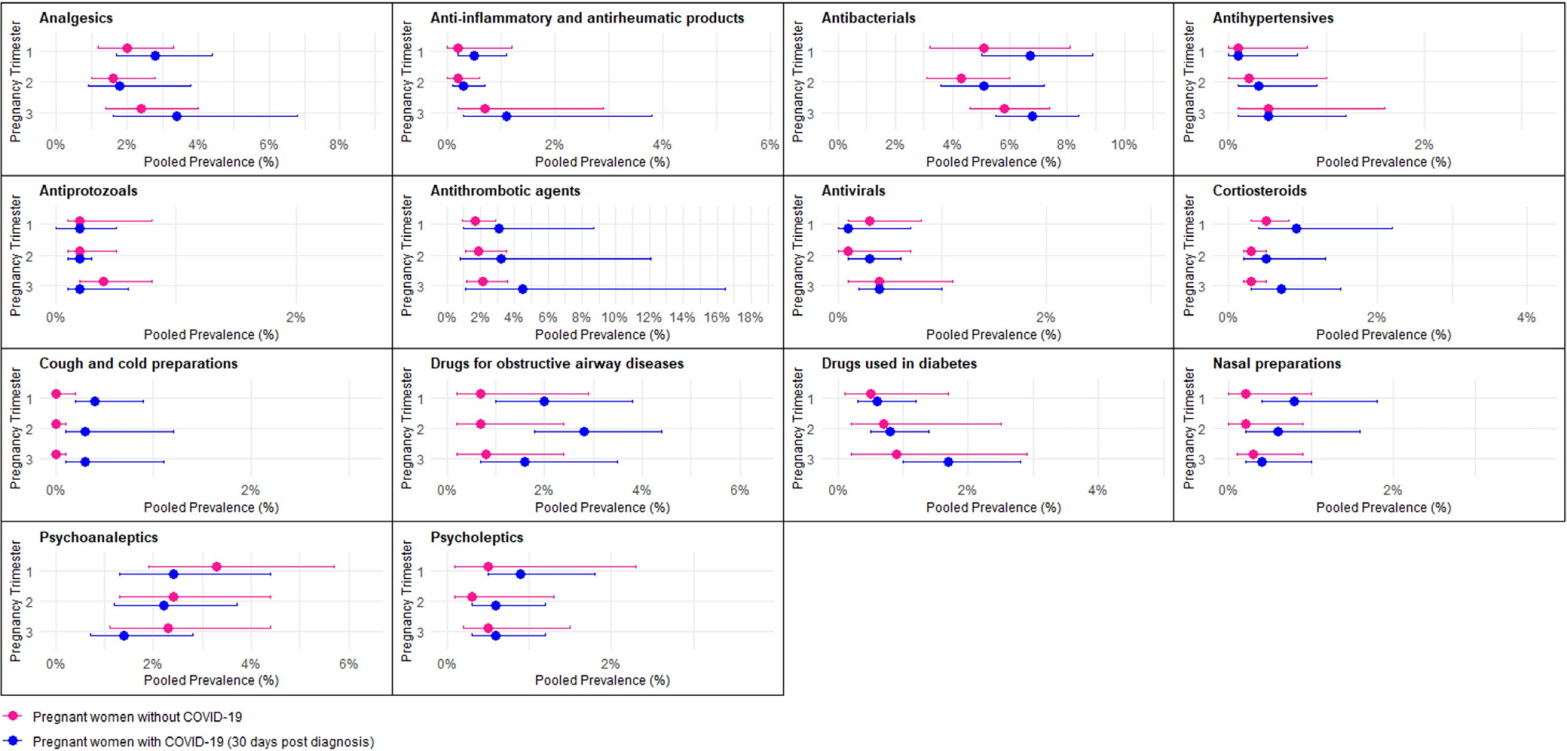
Pooled prevalence of medication use in the 30 days post‐COVID‐19 diagnosis in nonhospitalized pregnant women with COVID‐19 compared to pregnant women without COVID‐19 by medication group and pregnancy trimester.

The baseline characteristics of the cohorts of nonhospitalized pregnant women with and without COVID‐19 across the study sites are presented in Table S6. Figure 3 shows the pooled prevalence of medication use in the 30 days post‐COVID‐19 diagnosis in nonhospitalized pregnant women with COVID‐19 and pregnant women without COVID‐19 by medication group and pregnancy trimester. The corresponding forest plots showing the pooled prevalence of all medication groups are provided in Figures S1–S22 and indicate high heterogeneity in nearly all analyses. None of the pooled prevalence rates were significantly different between pregnant women with COVID‐19 and those without COVID‐19. However, pooled prevalence rates of the use of analgesics, anti‐inflammatory and antirheumatic products, antibacterials, antithrombotic agents, corticosteroids, cough and cold preparations, drugs for obstructive airway diseases, drugs used in diabetes, nasal preparations, and psycholeptics were generally higher across all trimesters in pregnant women with COVID‐19 compared with those without COVID‐19 (Figure 3).

The greatest difference in 30 days post‐COVID‐19 pooled prevalence of medication use between nonhospitalized pregnant women with and without COVID‐19 was observed for antithrombotic agents. In the third trimester, the pooled prevalence of antithrombotic agents 30 days post‐COVID‐19 was 4.5% (95% CI = 1.1–16.5, *I* ^2^ = 100%) in pregnant women with COVID‐19, compared to 2.1% (95% CI = 1.2–3.6, *I* ^2^ = 99%) in pregnant women without COVID‐19 in the third trimester (Figure S11). The higher prescription post‐COVID‐19 diagnosis of drugs for obstructive airway diseases observed among pregnant women with COVID‐19 than those without COVID‐19 was most prominent in the second trimester (2.8%, 95% CI = 1.8–4.4, *I* ^2^ = 80% and 0.7%, 95% CI = 0.2–2.4, *I* ^2^ = 96%, respectively) (Figure S15). Regarding drugs used in diabetes, the difference was most prominent in the third trimester with a pooled prevalence of 1.7% (95% CI = 1.0–2.8, *I* ^2^ = 80%) in pregnant women with COVID‐19 compared to 0.9% (95% CI = 0.2–2.9, *I* ^2^ = 99%) in those without COVID‐19 (Figure S16). In contrast, the pooled prevalence of psychoanaleptics use was lower post‐COVID‐19 across all trimesters in pregnant women with COVID‐19 than among those without COVID‐19 (Figure S21). Overall, sensitivity analysis, excluding Canadian sites, did not materially change the results (Table S8).

### 3.3 Medication Use in Pregnant Women With COVID‐19 and Nonpregnant Women With COVID‐19

**FIGURE 4:**
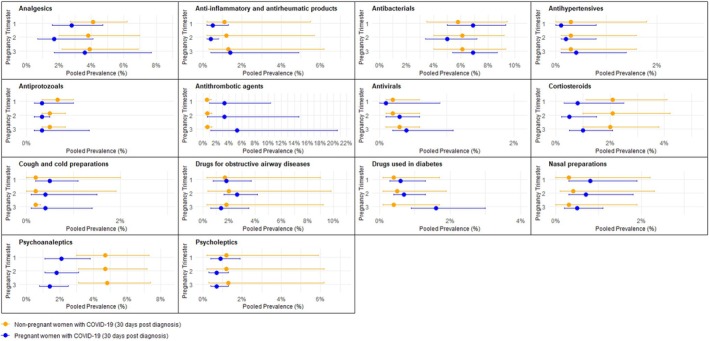
Pooled prevalence of medication use in the 30 days post‐COVID‐19 diagnosis in nonhospitalized pregnant women with COVID‐19 compared to nonpregnant women with COVID‐19 by medication group and pregnancy trimester.

The baseline characteristics of the cohorts of pregnant and nonpregnant women with COVID‐19 across the study sites are presented in Table S7. Wales, UK, did not have an adequate nonpregnant comparator group and was excluded from the analyses (Figure 1). Figure 4 shows the pooled prevalence of medication use in the 30 days post‐COVID‐19 diagnosis in nonhospitalized pregnant women with COVID‐19 compared to nonpregnant women with COVID‐19 by medication group and pregnancy trimester. The corresponding forest plots showing the pooled prevalence of all medication groups are provided in Figures S23–S44 and indicate high heterogeneity in nearly all analyses. Comparisons between the 30 days post‐COVID‐19 prevalence rates in pregnant women with COVID‐19 and nonpregnant women with COVID‐19 showed a statistically significant lower pooled prevalence of the use of psychoanaleptics in pregnant women with COVID‐19 in the third trimester (1.4%, 95% CI = 0.8–2.5, *I* ^2^ = 93%) compared to nonpregnant women with COVID‐19 (4.8%, 95% CI = 3.1–7.4, *I* ^2^ = 99%) (Figure S43). However, this difference was no longer statistically significant after excluding the Canadian cohort in the sensitivity analysis (Table S9). The pooled prevalence rates of the use of analgesics, antiprotozoals, corticosteroids, and psycholeptics were also generally lower across all trimesters in pregnant women with COVID‐19 compared with nonpregnant women with COVID‐19, although CIs overlapped (Figure 4). The lower prescription post‐COVID‐19 diagnosis of corticosteroids observed among pregnant women with COVID‐19 than nonpregnant women with COVID‐19 was most prominent in the second trimester (0.5%, 95% CI = 0.2–1.5, *I* ^2^ = 89% and 2.1%, 95% CI = 1.0–4.2, *I* ^2^ = 100%, respectively) (Figure S35).

In contrast, pooled prevalence rates of the use of antithrombotic agents, cough and cold preparations, drugs used in diabetes, and nasal preparations were generally higher across all trimesters in pregnant women with COVID‐19 compared with nonpregnant women with COVID‐19 (Figure 4). The greatest difference in 30 days post‐COVID‐19 pooled prevalence of medication use between nonhospitalized pregnant women with COVID‐19 and nonpregnant women with COVID‐19 was observed for antithrombotic agents. The 30 days post‐COVID‐19 pooled prevalence of antithrombotic agents was 5.2% (95% CI = 1.2–20.6, *I* ^2^ = 100%) in pregnant women with COVID‐19 in the third trimester compared with 0.7% (95% CI = 0.3–1.4, *I* ^2^ = 99%) in nonpregnant women with COVID‐19 (Figure S33). After excluding Canadian sites, the CIs for the pooled prevalence in all trimesters no longer overlapped (Table S9).

## 4 Discussion

This international prospective two‐stage IPD meta‐analysis, combining healthcare data from 10 data sources in seven countries, presents findings on medication utilization in a cohort of 50 335 pregnant women not hospitalized for their COVID‐19 event. Comparisons were made between the prevalence of medication use 30 days pre‐ and post‐COVID‐19 diagnosis in this cohort and in two comparator groups: pregnant women without COVID‐19 and nonpregnant women with COVID‐19.

Overall, the prevalence of medication use in pregnant women with COVID‐19 who were not hospitalized was low and varied widely across data sources. Among nonhospitalized pregnant women with COVID‐19, antibacterials, antithrombotic agents, and analgesics were the medications used most frequently, with antibacterials showing a statistically significant increase in dispensing during the third trimester in the 30 days following COVID‐19 diagnosis compared to the 30 days prior. The prevalence of medication use among nonhospitalized pregnant women with COVID‐19 was often higher than the prevalence among pregnant women without COVID‐19, although none of these differences reached statistical significance.

For some medications, the pooled prevalence was lower among nonhospitalized pregnant women with COVID‐19 compared with nonpregnant women with COVID‐19, for example, analgesics, antiprotozoals, corticosteroids, psychoanaleptics, and psycholeptics. Conversely, antithrombotic agents appeared to be more prevalent, although not significantly, among nonhospitalized pregnant women with COVID‐19 compared to nonpregnant women with COVID‐19. This finding likely reflects international guidelines recommending thromboprophylaxis for pregnant women with COVID‐19 [11]. Furthermore, in our meta‐analysis, the prevalence of antithrombotic agent use after a COVID‐19 diagnosis was notably higher in pregnant women with COVID‐19 than in pregnant women without COVID‐19, across all trimesters. This aligns with previous research indicating that pregnant women with COVID‐19 are at increased risk of developing venous thromboembolism compared to pregnant women without COVID‐19 [28, 29, 30]. Importantly, while our data did not allow us to distinguish between prophylactic and therapeutic use of antithrombotics, all medication records were captured in the outpatient setting. Since treatment for VTE generally requires hospitalization, we believe the increased use of antithrombotic agents most likely reflects outpatient prophylactic use. This interpretation is consistent with national guideline recommendations during the pandemic, and is further supported by the substantial geographic variation observed in antithrombotic prescribing. Post‐COVID‐19 diagnosis prevalence of antithrombotic use in pregnant women was highest in Spain, followed by Italy and Sweden, and was considerably lower in Canada, the United States, and Wales. This variation may be attributed to stronger recommendations for routine prophylaxis with low molecular weight heparin (LMWH) in European guidelines, while guidelines in the US recommended LMWH only for specific subgroups, for example, those with comorbidities [11].

Corticosteroids were mostly prescribed to nonhospitalized pregnant women in Italy and the United States, in line with national guidelines recommending their use for severe COVID‐19 cases from September 2020 and April 2020, respectively [11]. Compared to nonpregnant women, corticosteroids were less frequently prescribed to pregnant women. This may reflect caution due to safety concerns, but our data do not fully account for differences in baseline characteristics, and therefore, it is not possible to conclusively state that these nonhospitalized pregnant women were undertreated. The prescription rate of psychoanaleptics was markedly higher in Wales than elsewhere in Europe, as previously reported [31, 32]. The higher prevalence of drugs used in diabetes in pregnant women with COVID‐19 compared to nonpregnant women with COVID‐19 probably reflects gestational diabetes in the pregnant cohort. Additionally, our meta‐analysis highlights that anti‐inflammatory and antirheumatic products, which include NSAIDs, were prescribed in nonhospitalized pregnant women with and without COVID‐19 across all trimesters of pregnancy, particularly in the United States and Canada. However, NSAIDs are not recommended after 30 weeks of gestation due to their association with premature closure of the ductus arteriosus [33, 34]. Unfortunately, we could not identify the specific medication used, nor confirm whether these medications were prescribed for COVID‐19 treatment or obstetric management.

A systematic review and meta‐analysis of 62 case series and cohort studies published between December 2019 and February 2021 provided data on 31 016 pregnant women diagnosed with COVID‐19 [4]. The review indicated that among the studies reporting on the pharmacologic management of COVID‐19, approximately half of the pregnant women received antibiotics, anticoagulants, or hydroxychloroquine, while one in three were administered antivirals, and nearly one in five were managed with either corticosteroids or immunotherapy [6]. It is important to note that a key distinction in the studies included in that meta‐analysis was that most were conducted in a hospital setting, making direct comparisons with our study challenging. Additionally, results from the Observational Health Data Sciences and Informatics (OHDSI) network, which included electronic medical records and claims data from France, Spain, and the United States, and included 8598 pregnant women with COVID‐19, were focused on the inpatient settings [35]. Data from the outpatient setting were not available, making comparison with our findings impossible. The distinction between in and outpatient is important, as was already shown by previous studies within CONSIGN from the COVI‐PREG and INOSS initiatives [7, 9]. They showed that medication use is strongly associated with the severity of the disease, and therefore, the restriction to include nonhospitalized pregnant women with prescriptions in outpatient or primary care settings only is a key strength of our meta‐analysis. Another CONSIGN meta‐analysis is collecting information directly about medication use among hospitalized patients and should be referred to for that comparison [14].

Several limitations need to be considered when interpreting the findings of this meta‐analysis. First, we observed high levels of heterogeneity among the data sources, likely due to differences in health systems, prescription status, reimbursement practices, and clinical guidelines across countries. These factors may have influenced the availability, accessibility, and utilization of medications among pregnant women with COVID‐19, leading to the observed variation in prevalence rates. For example, in Canada, Alberta and Manitoba have pharmaceutical claims for the total population, whereas Ontario has very few people under 65 eligible for provincial drug reimbursement. In Wales, UK, some of the highest prescription rates were observed, possibly due to free access to medicines at the point of need. Data from the Sentinel System primarily included a commercially insured population, potentially underrepresenting publicly insured persons or uninsured persons. We were also unable to adjust for covariates such as socioeconomic status, substance misuse, and smoking, which were captured in some data sources but not in others. Further research examining the influence of health system structures on medication utilization during pregnancy and COVID‐19 infection will be valuable for understanding these heterogeneities.

Another potential explanation for heterogeneity is the variation in data collection methods and challenges in harmonizing data across sources. Despite significant efforts to share shell tables, definitions, and other information between the CONSIGN EHR study, CAMCCO, and the Sentinel System, complete alignment was not always possible. For instance, CAMCCO did not apply matching, though baseline characteristics were comparable. The Sentinel System includes only pregnancies resulting in live births, which may favor low‐risk pregnancies. Pregnancies at risk of miscarriage could have different medication use patterns, particularly in the first trimester. However, as Sentinel is the largest dataset, excluding it would substantially reduce the sample size. Differences in the study period, trimester definitions, and age groups, as well as distinct approaches to defining other variables, further complicated harmonization. Nonetheless, we pursued alignment as far as possible. It is also important to note that the meta‐analysis of proportions typically results in large *I* ^2^ values, which do not necessarily indicate inconsistent data [36].

Second, stratification by trimester and the restriction to nonhospitalized pregnant women led to small sample sizes in some data sources, resulting in less precise prevalence estimates, as evidenced by wide CIs. Additionally, due to the rule of reporting one event for data sources that were not allowed to share very low numbers, we may sometimes have underestimated the prevalence.

Third, although our study compared pre‐ and post‐COVID‐19 medication use in pregnant and nonpregnant cohorts, a difference‐in‐differences (DiD) analysis could have provided a more robust approach to account for baseline differences in medication use and comorbidities. Conducting a DiD analysis was not part of the study protocol and was further limited by the absence of matching in some datasets. We acknowledge this as a limitation while recommending DiD analyses as a promising approach for future research using similar international data.

Finally, conducting a multinational analysis across diverse healthcare systems and data sources presents several challenges. Large countries with more data can dominate the overall estimates, while differences in data collection methods, prescription practices, reimbursement policies, and study periods contribute to heterogeneity. Despite these limitations, pooling data allows for broader insights into medication use patterns and variation across countries. These considerations should be kept in mind when interpreting our findings, particularly regarding the generalizability of prevalence estimates.

## 5 Conclusion

The findings of this international prospective meta‐analysis, which includes 50 335 nonhospitalized pregnant women with COVID‐19 from seven countries and covers a considerable period spanning from January 2020 to December 2022, offer valuable insights into medication utilization patterns. The data provides rich information on the similarities and disparities across different countries. This information contributes significantly to a comprehensive understanding of COVID‐19 management strategies in nonhospitalized pregnant women, informing evidence‐based decision‐making in diverse healthcare settings.

## Author Contributions

The CONSIGN core research team (O.d.B., E.M., E.H., H.M.E.N., E.A., F.A., G.F., A.P., K.W.M.B., K.P., C.d.V., S.J.S., and M.C.J.M.S.) drafted the protocol and SAP. The protocol and SAP were revised and finalized based on feedback from all co‐authors. O.d.B. and E.M. coordinated contact with all data access providers. The following authors had the main responsibility for local data analysis: Tuscany, Italy (R.G. and O.P.), France (R.L. and M.‐A.B.), Valencia, Spain (C.L.R.‐B. and F.S.‐S.), Aragon, Spain (B.P.‐P.), Wales, UK (S.J. and D.T.), Norway (H.M.E.N.), Sweden (C.E.C. and D.H.), Canada (A.B., O.S., and P.K.), the United States (M.U.S., A.C., J.G.L., E.M.‐J., M.E.K., S.T., W.H., J.J.H.‐M., and L.S.). O.d.B. developed the R scripts for meta‐analysis and created the figures and tables for all the results. All authors participated in interpreting the data, reviewing the manuscript, and approving the final version. Each author accepts accountability for their part of the paper as published.

Benjamin P. Geisler; Mark Walker; Steven Hawken; Sasha Bernatsky; Sherif Eltonsy; Emma Hoffman; Andrew B. Petrone; Jolene Mosley; Jenice Ko; Claudia Bartolini; Giuseppe Roberto; Giorgio Limoncella; Anna Girardi; Giulia Hyeraci; Antonio Gimeno‐Miguel; Jonás Carmona‐Pírez; Antonio Poncel‐Falcó; Aida Moreno‐Juste; Alexandra Prados‐Torres; Ian Farr; Saira Ahmed; Ieuan Scanlon; Gabriel Sanfélix‐Gimeno; Isabel Hurtado; Anibal Garcia‐Sempere; Salvador Peiro; Jérémy Jové; Dunia Sakr; Cécile Droz‐Perroteau; David Baum; Hilde M. Engjom; Riera‐Arnau; Mònica Sabaté Gallego; Elena Ballarín Alins; Cristina Aguilera Martin; Melissa Kampman; Celline Brasil.

## Funding

The research leading to the results for Covid‐19 infectiOn aNd medicineS In preGnancy (CONSIGN) was conducted as part of the activities of the EU PE&PV (Pharmacoepidemiology and Pharmacovigilance) Research Network, which is a public academic partnership coordinated by Utrecht University (UU), the Netherlands. The project has received support from the European Medicines Agency (EMA) under the Framework service contract no. EMA/2018/28/PE and was scientifically coordinated by the University Medical Center Utrecht (UMCU). The content of this document expresses the opinion of the authors and may not be understood or quoted as being made on behalf of or reflecting the position of the EMA or one of its committees or working parties. Electronic healthcare data sources participating in the CONSIGN EHR study were partly funded by the EMA under the abovementioned Framework service contract. Each of the other participating sites in this meta‐analysis has its own funding to collect the data and generate the evidence. The Canadian Mother–Child (CAMCCO) Active Surveillance Initiative is a pan‐Canadian program on drug safety and efficacy in pregnancy funded by the Canadian Institutes of Health Research (CIHR) and the Canada Foundation for Innovation (CFI) and scientifically coordinated by CHU Sainte‐Justine in Montreal, Quebec, Canada. The Sentinel System is a US government initiative managed and funded by the U.S. Food and Drug Administration (FDA) and scientifically coordinated by the Harvard Pilgrim Health Care Institute. This publication reflects the views of the authors (J.H., L.S., and W.H.) and should not be construed to represent FDA's views or policies.

## Conflicts of Interest

C.E.C., D.H., F.A., K.W.M.B., and M.C.J.M.S. report participation in research studies funded by pharmaceutical companies, with all funds paid to the institution where they are employed (no personal fees). The other authors declare no conflicts of interest.

## Supporting information


**Data S1:** Supporting Information.

## Data Availability

The aggregated results from the individual data sources that support the findings of this study are available on request from the corresponding author. The individual‐level data in each data source are not publicly available due to privacy, governance, or ethical restrictions.
