# Supplementary material for: Medications Used Among Nonhospitalized Pregnant Women With COVID‐19: A Prospective Individual Patient Data Meta‐Analysis in Europe and North America
Source: Pharmacoepidemiol Drug Saf. 2025 Dec 25;35(1):e70303. doi: 10.1002/pds.70303 (PMC12741507; doi:10.1002/pds.70303)
Supplement: Supplementary file 1 — Data S1: Supporting Information. [file PDS-35-e70303-s001.docx]

**SUPPLEMENTARY INFORMATION**

**Medications used among Non-Hospitalized Pregnant Women with COVID-19: a Prospective Individual Patient Data Meta-analysis in Europe and North America**

Odette de Bruin^1,2^, Emeline Maisonneuve^3,4,5^, Eimir Hurley^6^, Hedvig ME Nordeng^6,7^, Anick Bérard^8,9^, Odile Sheehy^8,9^, Padma Kaul^10^, Mayura U Shinde^11^, Austin Cosgrove^12^, Jennifer G Lyons^11^, Elizabeth Messenger-Jones^12^, Maria E Kempner^12^, Sengwee Toh^11^, Wei Hua^13^, José J Hernández-Muñoz^13^, Leyla Sahin^14^, Carolyn E Cesta^15^, David Hägg^15^, Rosa Gini^16^, Olga Paoletti^16^, Beatriz Poblador-Plou^17,18^, Sue Jordan^19^, Daniel Thayer^19^, Clara L Rodríguez-Bernal^18,20^, Francisco Sánchez-Sáez^18,20^, Régis Lassalle^21^, Marie-Agnès Bernard^21^, Ema Alsina^1^, Fariba Ahmadizar^1^, Guillaume Favre^5^, Alice Panchaud^3,5,22^, Kitty WM Bloemenkamp^2^, Kelly Plueschke^23^, Corinne de Vries^23^, Satu J Siiskonen^24^, and Miriam CJM Sturkenboom^1^, on behalf of the CONSIGN collaboration group^*^

^1^Department of Data Science & Biostatistics, Julius Global Health, University Medical Center Utrecht (UMCU), Utrecht, the Netherlands

^2^Department of Obstetrics, Division Woman and Baby, Wilhelmina Children’s Hospital, University Medical Center Utrecht (UMCU), Utrecht, the Netherlands

^3^Institute of Primary Health Care (BIHAM), University of Bern, Bern, Switzerland

^4^Graduate School for Health Sciences (GHS), University of Bern, Bern, Switzerland

^5^Materno-Fetal and Obstetrics Research Unit, Woman-Mother-Child Department, Lausanne University Hospital, Lausanne, Switzerland

^6^Pharmacoepidemiology and Drug Safety Research Group, Department of Pharmacy, University Oslo (UiO), Oslo, Norway

^7^Department of Child Health and Development, Norwegian Institute of Public Health, Oslo, Norway

^8^Faculty of Pharmacy, University of Montreal, Montreal, Quebec, Canada

^9^Centre Hospitalier Universitaire (CHU) de Sainte-Justine, Montreal, Quebec, Canada

^10^University of Alberta, Edmonton, Alberta, Canada

^11^Department of Population Medicine, Harvard Medical School and Harvard Pilgrim Health Care Institute, Boston, Massachusetts, USA

^12^Department of Population Medicine, Harvard Pilgrim Health Care Institute, Boston, Massachusetts, USA

^13^Office of Surveillance and Epidemiology, Center for Drug Evaluation and Research, U.S. Food and Drug Administration, Silver Spring, USA

^14^Office of New Drugs, Center for Drug Evaluation and Research, U.S. Food and Drug Administration, Silver Spring, USA

^15^Department of Medicine Solna, Centre for Pharmacoepidemiology, Karolinska Institutet, Stockholm, Sweden

^16^Tuscan Regional Healthcare Agency, Florence, Italy

^17^EpiChron Research Group, Aragon Health Sciences Institute (IACS), IIS Aragón, Miguel Servet University Hospital, Zaragoza, Spain

^18^Network for Research on Chronicity, Primary Care and Health Promotion (RICAPPS), Research Network on Health Services in Chronic Diseases, Institute of Health Carlos III, Madrid, Spain

^19^Faculty of Medicine, Health and Life Science, Swansea University, Swansea, Wales, UK

^20^Health Services Research and Pharmacoepidemiology Unit, Foundation for the Promotion of Health and Biomedical Research of Valencia Region, Valencia, Spain

^21^Bordeaux PharmacoEpi, INSERM CIC-P1401, Université de Bordeaux, Bordeaux, France

^22^Service of Pharmacy, Lausanne University Hospital and University of Lausanne, Lausanne, Switzerland

^23^European Medicines Agency, Amsterdam, the Netherlands

^24^Division of Pharmacoepidemiology and Clinical Pharmacology, Utrecht Institute for Pharmaceutical Sciences (UIPS), Utrecht University, Utrecht, the Netherlands

^*^Collaborators of CONSIGN are listed in Table S1

**TABLE OF CONTENTS**

[**Table S1.** Members of the CONSIGN collaboration group 5](#_Toc161298091)

[**Table S2.** Description of the data sources included in the meta-analysis 8](#_Toc161298092)

[**Table S3.** Data source specific methods for determining pregnancy start and end, COVID-19 diagnosis, and medication use 9](#_Toc161298093)

[**Table S4.** Covariates of interest 10](#_Toc161298094)

[**Table S5.** Medication groups of special relevance to COVID-19 at ATC level 2 11](#_Toc161298095)

[**Table S6.** Baseline characteristics of pregnant women with and without COVID-19 12](#_Toc161298096)

[**Figure S1.** Forest plots showing the pooled prevalence of analgesics in the 30 days pre-COVID (left) and 30 days post-COVID (right) in non-hospitalized pregnant women with COVID-19 (upper) and pregnant women without COVID-19 (lower), by pregnancy trimester 13](#_Toc161298097)

[**Figure S2.** Forest plots showing the pooled prevalence of anthelminthics in the 30 days pre-COVID (left) and 30 days post-COVID (right) in non-hospitalized pregnant women with COVID-19 (upper) and pregnant women without COVID-19 (lower), by pregnancy trimester 15](#_Toc161298098)

[**Figure S3.** Forest plots showing the pooled prevalence of anti-inflammatory and antirheumatic products in the 30 days pre-COVID (left) and 30 days post-COVID (right) in non-hospitalized pregnant women with COVID-19 (upper) and pregnant women without COVID-19 (lower), by pregnancy trimester 17](#_Toc161298099)

[**Figure S4.** Forest plots showing the pooled prevalence of antibacterials in the 30 days pre-COVID (left) and 30 days post-COVID (right) in non-hospitalized pregnant women with COVID-19 (upper) and pregnant women without COVID-19 (lower), by pregnancy trimester 19](#_Toc161298100)

[**Figure S5.** Forest plots showing the pooled prevalence of antigout preparations in the 30 days pre-COVID (left) and 30 days post-COVID (right) in non-hospitalized pregnant women with COVID-19 (upper) and pregnant women without COVID-19 (lower), by pregnancy trimester 21](#_Toc161298101)

[**Figure S6.** Forest plots showing the pooled prevalence of antihypertensives in the 30 days pre-COVID (left) and 30 days post-COVID (right) in non-hospitalized pregnant women with COVID-19 (upper) and pregnant women without COVID-19 (lower), by pregnancy trimester 23](#_Toc161298102)

[**Figure S7.** Forest plots showing the pooled prevalence of antimycobacterials in the 30 days pre-COVID (left) and 30 days post-COVID (right) in non-hospitalized pregnant women with COVID-19 (upper) and pregnant women without COVID-19 (lower), by pregnancy trimester 25](#_Toc161298103)

[**Figure S8.** Forest plots showing the pooled prevalence of antimycotics in the 30 days pre-COVID (left) and 30 days post-COVID (right) in non-hospitalized pregnant women with COVID-19 (upper) and pregnant women without COVID-19 (lower), by pregnancy trimester 27](#_Toc161298104)

[**Figure S9.** Forest plots showing the pooled prevalence of antineoplastic agents in the 30 days pre-COVID (left) and 30 days post-COVID (right) in non-hospitalized pregnant women with COVID-19 (upper) and pregnant women without COVID-19 (lower), by pregnancy trimester 29](#_Toc161298105)

[**Figure S10.** Forest plots showing the pooled prevalence of antiprotozoals in the 30 days pre-COVID (left) and 30 days post-COVID (right) in non-hospitalized pregnant women with COVID-19 (upper) and pregnant women without COVID-19 (lower), by pregnancy trimester 31](#_Toc161298106)

[**Figure S11.** Forest plots showing the pooled prevalence of antithrombotic agents in the 30 days pre-COVID (left) and 30 days post-COVID (right) in non-hospitalized pregnant women with COVID-19 (upper) and pregnant women without COVID-19 (lower), by pregnancy trimester 33](#_Toc161298107)

[**Figure S12.** Forest plots showing the pooled prevalence of antivirals in the 30 days pre-COVID (left) and 30 days post-COVID (right) in non-hospitalized pregnant women with COVID-19 (upper) and pregnant women without COVID-19 (lower), by pregnancy trimester 35](#_Toc161298108)

[**Figure S13.** Forest plots showing the pooled prevalence of corticosteroids in the 30 days pre-COVID (left) and 30 days post-COVID (right) in non-hospitalized pregnant women with COVID-19 (upper) and pregnant women without COVID-19 (lower), by pregnancy trimester 37](#_Toc161298109)

[**Figure S14.** Forest plots showing the pooled prevalence of cough and cold preparations in the 30 days pre-COVID (left) and 30 days post-COVID (right) in non-hospitalized pregnant women with COVID-19 (upper) and pregnant women without COVID-19 (lower), by pregnancy trimester 39](#_Toc161298110)

[**Figure S15.** Forest plots showing the pooled prevalence of drugs for obstructive airway diseases in the 30 days pre-COVID (left) and 30 days post-COVID (right) in non-hospitalized pregnant women with COVID-19 (upper) and pregnant women without COVID-19 (lower), by pregnancy trimester 41](#_Toc161298111)

[**Figure S16.** Forest plots showing the pooled prevalence of drugs used in diabetes in the 30 days pre-COVID (left) and 30 days post-COVID (right) in non-hospitalized pregnant women with COVID-19 (upper) and pregnant women without COVID-19 (lower), by pregnancy trimester 43](#_Toc161298112)

[**Figure S17.** Forest plots showing the pooled prevalence of immune sera and immunoglobulins in the 30 days pre-COVID (left) and 30 days post-COVID (right) in non-hospitalized pregnant women with COVID-19 (upper) and pregnant women without COVID-19 (lower), by pregnancy trimester 45](#_Toc161298113)

[**Figure S18.** Forest plots showing the pooled prevalence of immunostimulants in the 30 days pre-COVID (left) and 30 days post-COVID (right) in non-hospitalized pregnant women with COVID-19 (upper) and pregnant women without COVID-19 (lower), by pregnancy trimester 47](#_Toc161298114)

[**Figure S19.** Forest plots showing the pooled prevalence of immunosuppressants in the 30 days pre-COVID (left) and 30 days post-COVID (right) in non-hospitalized pregnant women with COVID-19 (upper) and pregnant women without COVID-19 (lower), by pregnancy trimester 49](#_Toc161298115)

[**Figure S20.** Forest plots showing the pooled prevalence of nasal preparations in the 30 days pre-COVID (left) and 30 days post-COVID (right) in non-hospitalized pregnant women with COVID-19 (upper) and pregnant women without COVID-19 (lower), by pregnancy trimester 51](#_Toc161298116)

[**Figure S21.** Forest plots showing the pooled prevalence of psychoanaleptics in the 30 days pre-COVID (left) and 30 days post-COVID (right) in non-hospitalized pregnant women with COVID-19 (upper) and pregnant women without COVID-19 (lower), by pregnancy trimester 53](#_Toc161298117)

[**Figure S22.** Forest plots showing the pooled prevalence of psycholeptics in the 30 days pre-COVID (left) and 30 days post-COVID (right) in non-hospitalized pregnant women with COVID-19 (upper) and pregnant women without COVID-19 (lower), by pregnancy trimester 55](#_Toc161298118)

[**Table S7.** Baseline characteristics of pregnant women with COVID-19 and non-pregnant women with COVID-19 57](#_Toc161298119)

[**Figure S23.** Forest plots showing the pooled prevalence of analgesics in the 30 days pre-COVID (left) and 30 days post-COVID (right) in non-hospitalized pregnant women with COVID-19 (upper) and non-hospitalized non-pregnant women with COVID-19 (lower), by pregnancy trimester 58](#_Toc161298120)

[**Figure S24.** Forest plots showing the pooled prevalence of anthelminthics in the 30 days pre-COVID (left) and 30 days post-COVID (right) in non-hospitalized pregnant women with COVID-19 (upper) and non-hospitalized non-pregnant women with COVID-19 (lower), by pregnancy trimester 60](#_Toc161298121)

[**Figure S25.** Forest plots showing the pooled prevalence of anti-inflammatory and antirheumatic products in the 30 days pre-COVID (left) and 30 days post-COVID (right) in non-hospitalized pregnant women with COVID-19 (upper) and non-hospitalized non-pregnant women with COVID-19 (lower), by pregnancy trimester 62](#_Toc161298122)

[**Figure S26.** Forest plots showing the pooled prevalence of antibacterials in the 30 days pre-COVID (left) and 30 days post-COVID (right) in non-hospitalized pregnant women with COVID-19 (upper) and non-hospitalized non-pregnant women with COVID-19 (lower), by pregnancy trimester 64](#_Toc161298123)

[**Figure S27.** Forest plots showing the pooled prevalence of antigout preparations in the 30 days pre-COVID (left) and 30 days post-COVID (right) in non-hospitalized pregnant women with COVID-19 (upper) and non-hospitalized non-pregnant women with COVID-19 (lower), by pregnancy trimester 66](#_Toc161298124)

[**Figure S28.** Forest plots showing the pooled prevalence of antihypertensives in the 30 days pre-COVID (left) and 30 days post-COVID (right) in non-hospitalized pregnant women with COVID-19 (upper) and non-hospitalized non-pregnant women with COVID-19 (lower), by pregnancy trimester 68](#_Toc161298125)

[**Figure S29.** Forest plots showing the pooled prevalence of antimycobacterials in the 30 days pre-COVID (left) and 30 days post-COVID (right) in non-hospitalized pregnant women with COVID-19 (upper) and non-hospitalized non-pregnant women with COVID-19 (lower), by pregnancy trimester 70](#_Toc161298126)

[**Figure S30.** Forest plots showing the pooled prevalence of antimycotics in the 30 days pre-COVID (left) and 30 days post-COVID (right) in non-hospitalized pregnant women with COVID-19 (upper) and non-hospitalized non-pregnant women with COVID-19 (lower), by pregnancy trimester 72](#_Toc161298127)

[**Figure S31.** Forest plots showing the pooled prevalence of antineoplastic agents in the 30 days pre-COVID (left) and 30 days post-COVID (right) in non-hospitalized pregnant women with COVID-19 (upper) and non-hospitalized non-pregnant women with COVID-19 (lower), by pregnancy trimester 74](#_Toc161298128)

[**Figure S32.** Forest plots showing the pooled prevalence of antiprotozoals in the 30 days pre-COVID (left) and 30 days post-COVID (right) in non-hospitalized pregnant women with COVID-19 (upper) and non-hospitalized non-pregnant women with COVID-19 (lower), by pregnancy trimester 76](#_Toc161298129)

[**Figure S33.** Forest plots showing the pooled prevalence of antithrombotic agents in the 30 days pre-COVID (left) and 30 days post-COVID (right) in non-hospitalized pregnant women with COVID-19 (upper) and non-hospitalized non-pregnant women with COVID-19 (lower), by pregnancy trimester 78](#_Toc161298130)

[**Figure S34.** Forest plots showing the pooled prevalence of antivirals in the 30 days pre-COVID (left) and 30 days post-COVID (right) in non-hospitalized pregnant women with COVID-19 (upper) and non-hospitalized non-pregnant women with COVID-19 (lower), by pregnancy trimester 80](#_Toc161298131)

[**Figure S35.** Forest plots showing the pooled prevalence of corticosteroids in the 30 days pre-COVID (left) and 30 days post-COVID (right) in non-hospitalized pregnant women with COVID-19 (upper) and non-hospitalized non-pregnant women with COVID-19 (lower), by pregnancy trimester 82](#_Toc161298132)

[**Figure S36.** Forest plots showing the pooled prevalence of cough and cold preparations in the 30 days pre-COVID (left) and 30 days post-COVID (right) in non-hospitalized pregnant women with COVID-19 (upper) and non-hospitalized non-pregnant women with COVID-19 (lower), by pregnancy trimester 84](#_Toc161298133)

[**Figure S37.** Forest plots showing the pooled prevalence of drugs for obstructive airway diseases in the 30 days pre-COVID (left) and 30 days post-COVID (right) in non-hospitalized pregnant women with COVID-19 (upper) and non-hospitalized non-pregnant women with COVID-19 (lower), by pregnancy trimester 86](#_Toc161298134)

[**Figure S38.** Forest plots showing the pooled prevalence of drugs used in diabetes in the 30 days pre-COVID (left) and 30 days post-COVID (right) in non-hospitalized pregnant women with COVID-19 (upper) and non-hospitalized non-pregnant women with COVID-19 (lower), by pregnancy trimester 88](#_Toc161298135)

[**Figure S39.** Forest plots showing the pooled prevalence of immune sera and immunoglobulins in the 30 days pre-COVID (left) and 30 days post-COVID (right) in non-hospitalized pregnant women with COVID-19 (upper) and non-hospitalized non-pregnant women with COVID-19 (lower), by pregnancy trimester 90](#_Toc161298136)

[**Figure S40.** Forest plots showing the pooled prevalence of immunostimulants in the 30 days pre-COVID (left) and 30 days post-COVID (right) in non-hospitalized pregnant women with COVID-19 (upper) and non-hospitalized non-pregnant women with COVID-19 (lower), by pregnancy trimester 92](#_Toc161298137)

[**Figure S41.** Forest plots showing the pooled prevalence of immunosuppressants in the 30 days pre-COVID (left) and 30 days post-COVID (right) in non-hospitalized pregnant women with COVID-19 (upper) and non-hospitalized non-pregnant women with COVID-19 (lower), by pregnancy trimester 94](#_Toc161298138)

[**Figure S42.** Forest plots showing the pooled prevalence of nasal preparations in the 30 days pre-COVID (left) and 30 days post-COVID (right) in non-hospitalized pregnant women with COVID-19 (upper) and non-hospitalized non-pregnant women with COVID-19 (lower), by pregnancy trimester 96](#_Toc161298139)

[**Figure S43.** Forest plots showing the pooled prevalence of psychoanaleptics in the 30 days pre-COVID (left) and 30 days post-COVID (right) in non-hospitalized pregnant women with COVID-19 (upper) and non-hospitalized non-pregnant women with COVID-19 (lower), by pregnancy trimester 98](#_Toc161298140)

[**Figure S44.** Forest plots showing the pooled prevalence of psycholeptics in the 30 days pre-COVID (left) and 30 days post-COVID (right) in non-hospitalized pregnant women with COVID-19 (upper) and non-hospitalized non-pregnant women with COVID-19 (lower), by pregnancy trimester 100](#_Toc161298141)

# **Table S1.** Members of the CONSIGN collaboration group

| **Organisation** | **Name** | **Affiliation** | **ORCID ID** |
| --- | --- | --- | --- |
| University of Oslo (CONSIGN WP1) | Benjamin P. Geisler | Pharmacoepidemiology and Drug Safety Research Group, Department of Pharmacy, University Oslo (UiO), Oslo, Norway | 0000-0003-1704-6067 |
| CAMCCO | Mark Walker | University of Ottawa, Ottawa, Ontario, Canada | 0000-0001-8974-4548 |
| CAMCCO | Steven Hawken | University of Ottawa, Ottawa, Ontario, Canada | 0000-0002-3341-9022 |
| CAMCCO | Sasha Bernatsky | Department of Epidemiology, Biostatistics and Occupational Health, McGill University, Montreal, Quebec, Canada | 0000-0002-9515-2802 |
| CAMCCO | Sherif Eltonsy | University of Manitoba, Winnipeg, Manitoba, Canada | 0000-0002-0520-5406 |
| Sentinel System | Emma Hoffman | Department of Population Medicine, Harvard Pilgrim Health Care Institute, Boston, Massachusetts, USA | N.A. |
| Sentinel System | Andrew B. Petrone | Department of Population Medicine, Harvard Pilgrim Health Care Institute, Boston, Massachusetts, USA | 0000-0001-8413-6236 |
| Sentinel System | Jolene Mosley | Department of Population Medicine, Harvard Pilgrim Health Care Institute, Boston, Massachusetts, USA | N.A. |
| Sentinel System | Jenice Ko | Department of Population Medicine, Harvard Pilgrim Health Care Institute, Boston, Massachusetts, USA | N.A. |
| ARS Toscana | Claudia Bartolini | Tuscan Regional Healthcare Agency, Florence, Italy | 0000-0001-8630-4598 |
| ARS Toscana | Giuseppe Roberto | Tuscan Regional Healthcare Agency, Florence, Italy | N.A. |
| ARS Toscana | Giorgio Limoncella | Tuscan Regional Healthcare Agency, Florence, Italy | N.A. |
| ARS Toscana | Anna Girardi | Tuscan Regional Healthcare Agency, Florence, Italy | N.A. |
| ARS Toscana | Giulia Hyeraci | Tuscan Regional Healthcare Agency, Florence, Italy | 0000-0002-6536-2083 |
| IACS | Antonio Gimeno-Miguel | EpiChron Research Group, Aragon Health Sciences Institute (IACS), IIS Aragón, Miguel Servet University Hospital, Zaragoza, Spain | 0000-0002-5440-1710 |
| IACS | Jonás Carmona-Pírez | EpiChron Research Group, Aragon Health Sciences Institute (IACS), IIS Aragón, Miguel Servet University Hospital, Zaragoza, Spain | 0000-0002-6268-8803 |
| IACS | Antonio Poncel-Falcó | EpiChron Research Group, Aragon Health Sciences Institute (IACS), IIS Aragón, Miguel Servet University Hospital, Zaragoza, Spain | N.A. |
| IACS | Aida Moreno-Juste | EpiChron Research Group, Aragon Health Sciences Institute (IACS), IIS Aragón, Miguel Servet University Hospital, Zaragoza, Spain | 0000-0002-8819-3278 |
| IACS | Alexandra Prados-Torres | EpiChron Research Group, Aragon Health Sciences Institute (IACS), IIS Aragón, Miguel Servet University Hospital, Zaragoza, Spain | 0000-0002-5704-6056 |
| SWANSEA | Ian Farr | Faculty of Medicine, Health and Life Science, Swansea University, Wales, UK | N.A. |
| SWANSEA | Saira Ahmed | Faculty of Medicine, Health and Life Science, Swansea University, Wales, UK | N.A. |
| SWANSEA | Ieuan Scanlon | Faculty of Medicine, Health and Life Science, Swansea University, Wales, UK | N.A. |

**Table S1 continued.** Members of the CONSIGN collaboration group

| **Organisation** | **Name** | **Affiliation** | **ORCID ID** |
| --- | --- | --- | --- |
| FISABIO-HSRU | Gabriel Sanfélix-Gimeno | Health Services Research and Pharmacoepidemiology Unit, Foundation for the Promotion of Health and Biomedical Research of Valencia Region, Valencia, Spain | 0000-0001-7098-4576 |
| FISABIO-HSRU | Isabel Hurtado | Health Services Research and Pharmacoepidemiology Unit, Foundation for the Promotion of Health and Biomedical Research of Valencia Region, Valencia, Spain | 0000-0002-8475-8112 |
| FISABIO-HSRU | Anibal Garcia-Sempere | Health Services Research and Pharmacoepidemiology Unit, Foundation for the Promotion of Health and Biomedical Research of Valencia Region, Valencia, Spain | N.A. |
| FISABIO-HSRU | Salvador Peiro | Health Services Research and Pharmacoepidemiology Unit, Foundation for the Promotion of Health and Biomedical Research of Valencia Region, Valencia, Spain | 0000-0002-3902-569X |
| BPE | Jérémy Jové | Bordeaux PharmacoEpi, INSERM CIC-P1401, Université de Bordeaux, Bordeaux, France | N.A. |
| BPE | Dunia Sakr | Bordeaux PharmacoEpi, INSERM CIC-P1401, Université de Bordeaux, Bordeaux, France | N.A. |
| BPE | Cécile Droz-Perroteau | Bordeaux PharmacoEpi, INSERM CIC-P1401, Université de Bordeaux, Bordeaux, France | 0000-0002-7697-1167 |
| COVI-PREG  (CONSIGN WP2) | David Baum | Materno-Fetal and Obstetrics Research Unit, Woman-Mother-Child Department, Lausanne University Hospital, Lausanne, Switzerland | N.A. |
| INOSS  (CONSIGN WP3) | Hilde M. Engjom | Department for Health Promotion and Department for Health Registry Research and Development, Norwegian Institute of Public Health, Bergen, Norway | 0000-0003-1582-4283 |
| Vall d’Hebron & UMC Utrecht | Riera-Arnau | Department of Clinical Pharmacology, Vall d'Hebron Hospital Universitari, Vall Hebron Institut de Recerca Barcelona, Spain & Department of Data Science & Biostatistics, Julius Global Health, University Medical Center Utrecht (UMCU), Utrecht, the Netherlands | 0000-0001-7591-0218 |
| Vall d’Hebron | Mònica Sabaté Gallego | Department of Clinical Pharmacology, Vall d'Hebron Hospital Universitari, Vall Hebron Institut de Recerca Barcelona, Spain | 0000-0001-6206-1085 |
| Vall d’Hebron | Elena Ballarín Alins | Department of Clinical Pharmacology, Vall d'Hebron Hospital Universitari, Vall Hebron Institut de Recerca Barcelona, Spain | 0000-0001-9786-6617 |
| Vall d’Hebron | Cristina Aguilera Martin | Department of Clinical Pharmacology, Vall d'Hebron Hospital Universitari, Vall Hebron Institut de Recerca Barcelona, Spain | 0000-0002-7985-7327 |
| Health Canada | Melissa Kampman | Data Analytics and Real-world Evidence Division, Health Products and Food Branch, Health Canada, Ottawa, Ontario, Canada | N.A. |
| Health Canada | Celline Brasil | Data Analytics and Real-world Evidence Division, Health Products and Food Branch, Health Canada, Ottawa, Ontario, Canada | N.A. |

N.A. = not available.

# **Table S2.** Description of the data sources for which individual-level data were provided

| **Data access provider**  **(data source)** | **Country**  **(area)** | **Estimated births per year** | **Type of data source** | **Medical birth registry** | **Diagnosis** | **Data availability** |
| --- | --- | --- | --- | --- | --- | --- |
| **CONSIGN EHR study** | | | | | | |
| ARS Toscana  (ARS database) | Italy (Tuscany) | 25 000 | Record linkage | Yes | In-hospital, emergency room | Mar 2020 – Dec 2021 |
| BPE  (SNDS) | France (national) | 700 000 | Health insurance | No | In-hospital | Mar 2020 – Dec 2020 |
| FISABIO-HSRU  (VID) | Spain (Valencia) | 32 000 | Record linkage | Yes | GP, In-hospital, Outpatient Specialists | Mar 2020 – Dec 2021 |
| IACS  (PRECOVID study aNd EpiChron Cohort) | Spain (Aragon) | 10 000 | Record linkage | Yes | GP, In-hospital | Mar 2020 – Dec 2021 |
| SWANSEA  (SAIL) | UK (Wales) | 33 000 | Record linkage | Yes | GP, In-hospital | Mar 2020 – Dec 2021 |
| UiO  (Linked national registries) | Norway (national) | 60 000 | Record linkage | Yes | GP, In-hospital | Mar 2020 – Dec 2021 |
| Karolinska Institutet  (Linked national registers) | Sweden (national) | 100 000 | Record linkage | Yes | In-hospital, Outpatient Specialists | Mar 2020 – Dec 2020 |
| **Total estimated births Europe:** | | **960 000 births/year** | | | | |
| **CAMCCO** | | | | | | |
| Alberta  (Linked databases) | Canada (Alberta) | 49 000 | Record linkage | Yes | GP, In-hospital, Outpatient Specialists, COVID-19 database | Mar 2020 - Aug 2021 |
| Manitoba  (Linked databases) | Canada (Manitoba) | 16 000 | Record linkage | Yes | GP, In-hospital, Outpatient Specialists, COVID-19 database | Mar 2020 - Feb 2021 |
| Ontario  (Linked databases) | Canada (Ontario) | 136 000 | Record linkage | Yes | GP, In-hospital, Outpatient Specialists, COVID-19 database | Mar 2020 - June 2021 |
| **Total estimated births in Canada:** | | **201 000 births/year** | | | | |
| **Sentinel System** | | | | | | |
| Sentinel System  (Sentinel Distributed Database) | US (national and regional coverage in Colorado, Oregon, Minnesota, and Washington States) | 485 000 | Health insurance | No | In-Hospital, Outpatient  Specialists | Jan 2020 - Dec 2022 |
| **Total estimated births in US:** | | **485 000 births/year** | | | | |
| **Total estimated births:** | | **1 685 000 births/year** | | | | |

Abbreviations: ARS = Agenzia Regionale di Sanita’ della Toscana; BPE = Bordeaux PharmacoEpi platform; CAMCCO = Canadian Mother-Child Cohort; EHR = electronic health record; FISABIO-HSRU = Foundation for the Promotion of Health and Biomedical Research of Valencia Region - Health Services Research Unit; GP: general practitioner, primary care; IACS = Instituto Aragones de Ciencias de la Salud; SAIL = Secure Anonymised Information Linkage; SNDS = Système National des Données de Santé; SWANSEA = Swansea University; UiO = University of Oslo; VID = Valencia Integrated Database.

# **Table S3.** Data source specific methods for determining pregnancy start and end, COVID-19 diagnosis, and medication use

| **DAP (data source)** | **Pregnancy start** | **Pregnancy end** | **COVID-19 diagnosis** | **Medications** |
| --- | --- | --- | --- | --- |
| **CONSIGN EHR study** | | | | |
| ARS Toscana (ARS database) | Date of record minus gestational age OR estimated on diagnostic code or period of pregnancy when a procedure is first expected | Date of record OR  estimated from due  date based on estimation of start date | Registry of positive COVID-19 tests (official surveillance system) | Outpatient  dispensing |
| BPE  (SNDS) | Date estimated from pregnancy algorithm which includes LMP and gestational age | Date of delivery | Inpatient data (PMSI) with ICD10 codes for COVID-19 diagnoses; no laboratory positive test result available. | Out + inpatient  dispensing |
| FISABIO-HSRU  (VID) | Date of delivery/ end of pregnancy minus gestational age (based on ultrasound/LMP) (95% pregnancies) OR Date of delivery minus 40 weeks when new-borns weight is 2500 gr or above and through a linear model when weight <2500 gr | Date of delivery or  abortion | All PCR or antigen test results are recorded in RedMIVA (Microbiological Surveillance Network of the Valencian Community) | Outpatient  dispensing |
| IACS  (PRECOVID study aNd EpiChron Cohort) | Date of the LMP | Date of the pregnancy  delivery or abortion recorded in primary care or hospital discharge | Registry developed for monitoring the evolution of COVID-19 disease in the region, includes all PCR or antigen test results | Outpatient  dispensing |
| SWANSEA  (SAIL) | Date of delivery (Monday of the week of birth as part of the irrevocable anonymisation process) minus gestational age at birth | Monday of the infant’s  week of delivery (to avoid use of identifiable data) | COVID-19 test results dataset available from healthdatagateay,org which include all test results and symptom trackers | Primary care |
| UiO  (Linked national registries) | Date of delivery minus gestational length in days based on ultrasound or LMP | Date of delivery (all pregnancies > gestational week 12, live or non-live) | Laboratory confirmed positive test recorded in MSIS (Norwegian surveillance system for communicable  diseases) | Outpatient  dispensing |
| Karolinska Institutet  (Linked national registries) | Date of delivery minus  gestational length in days  based on ultrasound or LMP | Date of delivery | All positive PCR test results registered in SmiNet (The Infectious Disease Register) and ICD-10 diagnosis for COVID-19 (National Patient register) | Outpatient  dispensing |
| **CAMCCO** | | | | |
| Alberta  (Linked databases) | First day of LMP (for deliveries) using gestational age reported in the delivery hospitalization chart summary AND algorithm to estimate first day of LMP (for spontaneous and planned abortions) | Clinically detected  spontaneous or  induced/planned abortion or  delivery | PCR test | Outpatient dispensing |
| Manitoba  (Linked databases) | First day of LMP (for deliveries) using gestational age reported in the delivery hospitalization chart summary AND algorithm to estimate first day of LMP (for spontaneous and planned abortions) | Clinically detected  spontaneous or  induced/planned abortion; or  delivery | PCR test | Outpatient dispensing |
| Ontario  (Linked databases) | First day of LMP (for deliveries) using gestational age reported in the delivery hospitalization chart summary AND algorithm to estimate first day of LMP (for spontaneous and planned abortions) | Clinically detected  spontaneous or  induced/planned abortion; or  delivery | PCR test | Outpatient dispensing  and some  in-patient  collected at  delivery |
| **Sentinel System** | | | | |
| Sentinel System  (Sentinel Distributed Database) | Estimated based on date of live-birth delivery and gestational age codes surrounding delivery date | ICD-10 diagnosis code  indicating live-birth  delivery | ICD-10 diagnosis code for  COVID-19 and/or positive  COVID-19 NAAT test | Outpatient dispensing |

Abbreviations: ARS = Agenzia Regionale di Sanita’ della Toscana; BPE = Bordeaux PharmacoEpi platform; CAMCCO = Canadian Mother-Child Cohort; EHR = electronic health record; FISABIO-HSRU = Foundation for the Promotion of Health and Biomedical Research of Valencia Region - Health Services Research Unit; IACS = Instituto Aragones de Ciencias de la Salud; LMP = last menstrual period; NAAT = Nucleic Acid Amplification Tests; PCR = polymerase chain reaction; SAIL = Secure Anonymised Information Linkage; SNDS = Système National des Données de Santé; SWANSEA = Swansea University; UiO = University of Oslo; VID = Valencia Integrated Database.

# **Table S4.** Covariates of interest

| **Covariate** | **Definition** | **Identification of covariate** | **Assessment window** |
| --- | --- | --- | --- |
| Age of mother | Categorized into three groups: 12-24 years of age, 25-39 years of age, 40-55 years of age in CONSIGN EHR study and Sentinel. In CAMCCO, the age groups are 15-24 years of age, 25-39 years of age, and 40-45 years of age. | Date of birth | T0 |
| Trimester of pregnancy | CONSIGN EHR: The ACOG definition of timing in pregnancy is used:   1. Trimester 1: from Last Menstrual Period (LMP) to day 97 after LMP; or end of pregnancy, whichever earlier 2. Trimester 2: from day 98 after LMP to day 195 after LMP; or end of pregnancy, whichever earlier 3. Trimester 3: from day 196 after LMP onwards until end of pregnancy   For Sentinel:   1. First trimester: days 0 to 90 of gestation (13 weeks) 2. Second trimester: days 91 to 180 (13+1 to 25+5 weeks) 3. Third trimester: days 181 (≥25+6 weeks through the day of the hospital admission for live-birth delivery.   For CAMCCO:   1. 1^st^ trimester: 0-98 days (> 0 to ≥14 weeks gestation) 2. 2^nd^ trimester: 99-182 days (>14 to ≥26 weeks’ gestation) 3. 3^rd^ trimester: ≥183 days (≥26+1 weeks’ gestation to end of the pregnancy) | Pregnancy algorithm | T0 |
| Covid-19 positive test or diagnosis | Recording of a positive COVID-19 test (PCR or antigen test), or presence of a diagnostic code in health care records or mandatory notification to the surveillance system | Recording of “COVID-19” in registry data OR diagnostic codes AND/OR laboratory results | T0 |
| COVID-19 severity | Non-hospitalized women had a recording of a positive COVID-19 test or diagnosis, with no subsequent hospital admission with a recording (primary or secondary) of COVID-19 (within a 4-week period). Hospitalized women had any recording of ‘COVID-19 positive test’, or ‘COVID-19 complication’ in any of the diagnostic fields in hospital records, not just the principal diagnosis. However, if the COVID-19 test positive test was two days of delivery date/hospitalization for obstetric reasons, and no codes of severe symptoms (pneumonia, respiratory aid/use of ventilator) were found subsequently, these women were excluded from the severe COVID-19 group. This was not conducted in Sentinel, where severe COVID-19 was defined as hospitalized patients with COVID-19 complications, ICU admission, ventilation, or death; and non-severe COVID-19 as diagnosis of COVID-19 or positive SARS-COV-2 test. |  | T0 |
| At-risk medical conditions for severe COVID-19 | At risk conditions for severe COVID-19 were divided into following subcategories:   - Cardiovascular disease/serious heart conditions include heart failure, coronary artery disease, cardiac myopathies - Hypertension - Sickle cell disease - Chronic lung disease including COPD, cystic fibrosis, severe asthma, interstitial lung disease, pulmonary hypertension, bronchiectasis - Type 1 & 2 Diabetes - Obesity diagnosis or having a BMI ≥30 kg/m2 - Chronic kidney disease - Chronic liver disease diagnosis (cirrhosis, non-alcoholic fatty liver disease, alcoholic liver disease, autoimmune hepatitis) - HIV - Common rheumatic diseases - Immunosuppression & solid organ transplants - Cancer - Mental health disease (depression, dementia, and schizophrenia spectrum disorders) | Recording of the at-risk medical conditions for severe COVID-19 OR diagnostic codes AND medicines proxies | Entire period of available data prior to T0 (including a minimum 1-year look-back period) |
| Risk conditions for obstetric complications | Prior maternal history of gestational diabetes or pre-eclampsia and prior history of stillbirth or late miscarriage, small for gestational age (SGA) child or foetal growth restriction (FGR), or child with congenital anomaly. | Recording of risk conditions for obstetric complications OR diagnostic codes | Entire period of available data prior to T0 |
| Calendar month of COVID-19 diagnosis | December 2019 till [according to each data source] | Date of diagnosis/positive test | T0 |

Abbreviations: ACOG = American College of Obstetricians and Gynaecologists; BMI = body mass index; CAMCCO = Canadian Mother-Child Cohort; EHR = electronic health record; LMP = last menstrual period; PCR = polymerase chain reaction.

# **Table S5.** Medication groups of special relevance to COVID-19 at ATC level 2

| **Name** | **Anatomical Therapeutic Classification (ATC) level 2** |
| --- | --- |
| Analgesics | N02 |
| Anthelminthics | P02 |
| Anti-inflammatory and antirheumatic products | M01 |
| Antibacterials for systemic use | J01 |
| Antigout preparations | M04 |
| Antihypertensives | C02, C03, C04, C07, C08 and/or C09 |
| Antimycobacterials | J04 |
| Antimycotics for systemic use | J02 |
| Antineoplastic agents | L01 |
| Antiprotozoals | P01 |
| Antithrombotic agents | B01 |
| Antivirals for systemic use | J05 |
| Corticosteroids for systemic use | H02 |
| Cough and cold preparations | R05 |
| Drugs for obstructive airway diseases | R03 |
| Drugs used in diabetes | A10 |
| Immune sera and immunoglobulins | J06 |
| Immunostimulants | L03 |
| Immunosuppressants | L04 |
| Nasal preparations | R01 |
| Psychoanaleptics | N06 |
| Psycholeptics | N05 |

# **Table S6.** Baseline characteristics of non-hospitalized pregnant women with and without COVID-19

| **Study site** | **Trimester at  COVID-19 infection** | **COVID-19 severity** | **Age** | | **Co-morbidities^1^** | | **Obstetric risk^2^** | |
| --- | --- | --- | --- | --- | --- | --- | --- | --- |
|  | **Pregnant with**  **COVID-19** | **Pregnant without COVID-19** | **Pregnant with**  **COVID-19** | **Pregnant without COVID-19** | **Pregnant with**  **COVID-19** | **Pregnant without COVID-19** | **Pregnant with**  **COVID-19** | **Pregnant without COVID-19** |
| Tuscany, Italy | 1^st^ trimester: 24.2%  2^nd^ trimester: 35.2%  3^rd^ trimester: 40.6% | 1^st^ trimester: 20.4%  2^nd^ trimester: 27.8%  3^rd^ trimester: 51.8% | 12-24 years: 10.7%  25-39 years: 82.6%  40-55 years: 6.7% | 12-24 years: 11.1%  25-39 years: 81.6%  40-55 years: 7.3% | Any: 21.3% Cardiovascular: 41.%  Chronic lung: 4.7%  Severe obesity: 0.3% | Any: 22.1% Cardiovascular: 4.2%  Chronic lung: 5.7%  Severe obesity: 0.2% | Any: 8.5% Adverse pregnancy outcomes: 7.1% | Any: 6.4% Adverse pregnancy outcomes: 5.2% |
| Valencia, Spain | 1^st^ trimester: 38.2%  2^nd^ trimester: 32.0%  3^rd^ trimester: 29.7% | 1^st^ trimester: 34.8%  2^nd^ trimester: 28.7%  3^rd^ trimester: 36.5% | 12-24 years: 12.6%  25-39 years: 77.2%  40-55 years: 10.2% | 12-24 years: 13.2%  25-39 years: 76.9%  40-55 years: 9.9% | Any: 24.2% Cardiovascular: 6.2%  Chronic lung: 10.1%  Severe obesity: 1.8% | Any: 21.5% Cardiovascular: 4.4%  Chronic lung: 9.0%  Severe obesity: 1.4% | Any: 12.0% Adverse pregnancy outcomes: 9.6% | Any: 7.1% Adverse pregnancy outcomes: 5.5% |
| Aragon, Spain | 1^st^ trimester: 25.2%  2^nd^ trimester: 35.8%  3^rd^ trimester: 39.0% | 1^st^ trimester: 20.6%  2^nd^ trimester: 29.0%  3^rd^ trimester: 50.3% | 12-24 years: 12.3%  25-39 years: 78.8%  40-55 years: 8.9% | 12-24 years: 13.9%  25-39 years: 78.3%  40-55 years: 7.8% | Any: 25.6% Cardiovascular: 4.6%  Chronic lung: 8.2%  Severe obesity: 0.4% | Any: 24.4% Cardiovascular: 3.3%  Chronic lung: 8.3%  Severe obesity: 0.6% | Any: 10.3% Adverse pregnancy outcomes: 8.2% | Any: 9.1% Adverse pregnancy outcomes: 7.0% |
| Wales, UK | 1^st^ trimester: 33.6%  2^nd^ trimester: 36.4%  3^rd^ trimester: 30.0% | 1^st^ trimester: 29.5%  2^nd^ trimester: 32.6%  3^rd^ trimester: 37.8% | 12-24 years: N.A.  25-39 years: N.A.  40-55 years: N.A. | 12-24 years: N.A.  25-39 years: N.A.  40-55 years: N.A. | Any: 29.6% Cardiovascular: 1.2%  Chronic lung: 10.0%  Severe obesity: 1.2% | Any: 28.5% Cardiovascular: 1.1%  Chronic lung: 9.5%  Severe obesity: 0.7% | Any: 18.4% Adverse pregnancy outcomes: N.A. | Any: 17.4% Adverse pregnancy outcomes: N.A. |
| Norway | 1^st^ trimester: 24.7%  2^nd^ trimester: 41.1%  3^rd^ trimester: 34.2% | 1^st^ trimester: 20.5%  2^nd^ trimester: 35.7%  3^rd^ trimester: 43.8% | 12-24 years: 10.4%  25-39 years: 87.1%  40-55 years: 2.5% | 12-24 years: 10.6%  25-39 years: 86.2%  40-55 years: 3.2% | Any: 18.5% Cardiovascular: 3.5%  Chronic lung: 4.3%  Severe obesity: 1.5% | Any: 21.5% Cardiovascular: 2.7%  Chronic lung: 5.8%  Severe obesity: 1.3% | Any: 2.0% Adverse pregnancy outcomes: 0.3% | Any: 1.7% Adverse pregnancy outcomes: 0.3% |
| Sweden | 1^st^ trimester: 6.4%  2^nd^ trimester: 35.4%  3^rd^ trimester: 58.2% | 1^st^ trimester: 5.9%  2^nd^ trimester: 34.6%  3^rd^ trimester: 59.4% | 12-24 years: 6.1%  25-39 years: 88.6%  40-55 years: 5.2% | 12-24 years: 6.2%  25-39 years: 88.5%  40-55 years: 5.3% | Any: 5.7% Cardiovascular: 0.4%  Chronic lung: 0.8%  Severe obesity: 3.3% | Any: 5.5% Cardiovascular: 0.6%  Chronic lung: 0.7%  Severe obesity: 3.0% | Any: N.A. Adverse pregnancy outcomes: N.A. | Any: N.A. Adverse pregnancy outcomes: N.A. |
| Alberta, Canada | 1^st^ trimester: 29.0%  2^nd^ trimester: 35.9%  3^rd^ trimester: 35.1% | 1^st^ trimester: 25.3%  2^nd^ trimester: 32.3%  3^rd^ trimester: 42.5% | 15-24 years: 14.8%  25-39 years: 81.9%  40-45 years: 3.3% | 12-24 years: 13.7%  25-39 years: 82.9%  40-55 years: 3.5% | Any: 41.4% Cardiovascular: 2.6%  Respiratory: 7.1%  Severe obesity: 1.8% | Any: 43.0% Cardiovascular: 2.7%  Respiratory: 9.0%  Severe obesity: 1.4% | Any: N.A. Adverse reproductive history outcomes: 5.5% | Any: N.A. Adverse reproductive history outcomes: 5.2% |
| Manitoba, Canada | 1^st^ trimester: 11.2%  2^nd^ trimester: 36.5%  3^rd^ trimester: 52.4% | 1^st^ trimester: 8.1%  2^nd^ trimester: 26.4%  3^rd^ trimester: 65.5% | 15-24 years: >3.5%  25-39 years: 70.6%  40-45 years: <3.5% | 12-24 years: 22.5%  25-39 years: 74.2%  40-55 years: 3.4% | Any: 36.5% Cardiovascular: <3.5%  Respiratory: >3.5%  Severe obesity: <3.5% | Any: 42.6% Cardiovascular: 1.4%  Respiratory: 9.2%  Severe obesity: 2.0% | Any: N.A. Adverse reproductive history outcomes: <3.5% | Any: N.A. Adverse reproductive history outcomes: 2.7% |
| Ontario, Canada | 1^st^ trimester: 28.9%  2^nd^ trimester: 32.5%  3^rd^ trimester: 38.6% | 1^st^ trimester: 28.9%  2^nd^ trimester: 32.5%  3^rd^ trimester: 38.6% | 15-24 years: 34.7%  25-39 years: 61.7%  40-45 years: 3.5% | 12-24 years: 34.8%  25-39 years: 61.2%  40-55 years: 4.0% | Any: 28.9% Cardiovascular: 0%  Respiratory: 2.6%  Severe obesity: 1.2% | Any: 33.8% Cardiovascular: 0.03%  Respiratory: 2.7%  Severe obesity: 1.6% | Any: N.A. Adverse reproductive history outcomes: 2.8% | Any: N.A. Adverse reproductive history outcomes: 3.5% |
| U.S. | 1^st^ trimester: 18.8%  2^nd^ trimester: 26.7%  3^rd^ trimester: 54.5% | 1^st^ trimester: 19.6%  2^nd^ trimester: 27.1%  3^rd^ trimester: 53.3% | 12-24 years: NA  25-39 years: NA  40-55 years: NA | 12-24 years: 16.7%  25-39 years: 79.9%  40-55 years: 3.4% | Any: N.A. Cardiovascular: 0.9%  Chronic lung: 6.3%  Severe obesity: 15.4% | Any: N.A. Cardiovascular: 0.8%  Chronic lung: 6.2%  Severe obesity: 15.4% | Any: N.A. Adverse pregnancy outcomes: 5.1% | Any: N.A. Adverse pregnancy outcomes: 5.0% |

^1^Any co-morbidities included: cancer, cardiovascular disease, chronic kidney disease, chronic liver disease, chronic lung disease, common rheumatic disease, diabetes, HIV, hypertension, mental disorders, severe obesity, sickle cell disease, and use of immunosuppressants.^2^Any obstetric risk included: prior history of the following: gestational diabetes, gestational hypertension, pre-eclampsia, HELLP and adverse pregnancy outcomes. Adverse pregnancy outcomes included: stillbirth, spontaneous abortion, SGA, FGR, and major congenital abnormalities. Abbreviations: N.A. = not available.

# **Figure S1.** Forest plots showing the pooled prevalence of analgesics in the 30 days pre-COVID (left) and 30 days post-COVID (right) in non-hospitalized pregnant women with COVID-19 (upper) and pregnant women without COVID-19 (lower), by pregnancy trimester

| **COVID-19 diagnosis in pregnancy trimester 1** | |
| --- | --- |
|  |  |
|  |  |
| **COVID-19 diagnosis in pregnancy trimester 2** | |
|  |  |
|  |  |

**Figure S1 continued.** Forest plots showing the pooled prevalence of analgesics in the 30 days pre-COVID (left) and 30 days post-COVID (right) in non-hospitalized pregnant women with COVID-19 (upper) and pregnant women without COVID-19 (lower), by pregnancy trimester

| **COVID-19 diagnosis in pregnancy trimester 3** | |
| --- | --- |
|  |  |
|  |  |

# **Figure S2.** Forest plots showing the pooled prevalence of anthelminthics in the 30 days pre-COVID (left) and 30 days post-COVID (right) in non-hospitalized pregnant women with COVID-19 (upper) and pregnant women without COVID-19 (lower), by pregnancy trimester

| **COVID-19 diagnosis in pregnancy trimester 1** | |
| --- | --- |
|  |  |
|  |  |
| **COVID-19 diagnosis in pregnancy trimester 2** | |
|  |  |
|  |  |

**Figure S2 continued.** Forest plots showing the pooled prevalence of anthelminthics in the 30 days pre-COVID (left) and 30 days post-COVID (right) in non-hospitalized pregnant women with COVID-19 (upper) and pregnant women without COVID-19 (lower), by pregnancy trimester

| **COVID-19 diagnosis in pregnancy trimester 3** | |
| --- | --- |
|  |  |
|  |  |

# **Figure S3.** Forest plots showing the pooled prevalence of anti-inflammatory and antirheumatic products in the 30 days pre-COVID (left) and 30 days post-COVID (right) in non-hospitalized pregnant women with COVID-19 (upper) and pregnant women without COVID-19 (lower), by pregnancy trimester

| **COVID-19 diagnosis in pregnancy trimester 1** | |
| --- | --- |
|  |  |
|  |  |
| **COVID-19 diagnosis in pregnancy trimester 2** | |
|  |  |
|  |  |

**Figure S3 continued.** Forest plots showing the pooled prevalence of anti-inflammatory and antirheumatic products in the 30 days pre-COVID (left) and 30 days post-COVID (right) in non-hospitalized pregnant women with COVID-19 (upper) and pregnant women without COVID-19 (lower), by pregnancy trimester

| **COVID-19 diagnosis in pregnancy trimester 3** | |
| --- | --- |
|  |  |
|  |  |

# **Figure S4.** Forest plots showing the pooled prevalence of antibacterials in the 30 days pre-COVID (left) and 30 days post-COVID (right) in non-hospitalized pregnant women with COVID-19 (upper) and pregnant women without COVID-19 (lower), by pregnancy trimester

| **COVID-19 diagnosis in pregnancy trimester 1** | |
| --- | --- |
|  |  |
|  |  |
| **COVID-19 diagnosis in pregnancy trimester 2** | |
|  |  |
|  |  |

**Figure S4 continued.** Forest plots showing the pooled prevalence of antibacterials in the 30 days pre-COVID (left) and 30 days post-COVID (right) in non-hospitalized pregnant women with COVID-19 (upper) and pregnant women without COVID-19 (lower), by pregnancy trimester

| **COVID-19 diagnosis in pregnancy trimester 3** | |
| --- | --- |
|  |  |
|  |  |

# **Figure S5.** Forest plots showing the pooled prevalence of antigout preparations in the 30 days pre-COVID (left) and 30 days post-COVID (right) in non-hospitalized pregnant women with COVID-19 (upper) and pregnant women without COVID-19 (lower), by pregnancy trimester

| **COVID-19 diagnosis in pregnancy trimester 1** | |
| --- | --- |
|  |  |
|  |  |
| **COVID-19 diagnosis in pregnancy trimester 2** | |
|  |  |
|  |  |

**Figure S5 continued.** Forest plots showing the pooled prevalence of antigout preparations in the 30 days pre-COVID (left) and 30 days post-COVID (right) in non-hospitalized pregnant women with COVID-19 (upper) and pregnant women without COVID-19 (lower), by pregnancy trimester

| **COVID-19 diagnosis in pregnancy trimester 3** | |
| --- | --- |
|  |  |
|  |  |

# **Figure S6.** Forest plots showing the pooled prevalence of antihypertensives in the 30 days pre-COVID (left) and 30 days post-COVID (right) in non-hospitalized pregnant women with COVID-19 (upper) and pregnant women without COVID-19 (lower), by pregnancy trimester

| **COVID-19 diagnosis in pregnancy trimester 1** | |
| --- | --- |
|  |  |
|  |  |
| **COVID-19 diagnosis in pregnancy trimester 2** | |
|  |  |
|  |  |

**Figure S6 continued.** Forest plots showing the pooled prevalence of antihypertensives in the 30 days pre-COVID (left) and 30 days post-COVID (right) in non-hospitalized pregnant women with COVID-19 (upper) and pregnant women without COVID-19 (lower), by pregnancy trimester

| **COVID-19 diagnosis in pregnancy trimester 3** | |
| --- | --- |
|  |  |
|  |  |

# **Figure S7.** Forest plots showing the pooled prevalence of antimycobacterials in the 30 days pre-COVID (left) and 30 days post-COVID (right) in non-hospitalized pregnant women with COVID-19 (upper) and pregnant women without COVID-19 (lower), by pregnancy trimester

| **COVID-19 diagnosis in pregnancy trimester 1** | |
| --- | --- |
|  |  |
|  |  |
| **COVID-19 diagnosis in pregnancy trimester 2** | |
|  |  |
|  |  |

**Figure S7 continued.** Forest plots showing the pooled prevalence of antimycobacterials in the 30 days pre-COVID (left) and 30 days post-COVID (right) in non-hospitalized pregnant women with COVID-19 (upper) and pregnant women without COVID-19 (lower), by pregnancy trimester

| **COVID-19 diagnosis in pregnancy trimester 3** | |
| --- | --- |
|  |  |
|  |  |

# **Figure S8.** Forest plots showing the pooled prevalence of antimycotics in the 30 days pre-COVID (left) and 30 days post-COVID (right) in non-hospitalized pregnant women with COVID-19 (upper) and pregnant women without COVID-19 (lower), by pregnancy trimester

| **COVID-19 diagnosis in pregnancy trimester 1** | |
| --- | --- |
|  |  |
|  |  |
| **COVID-19 diagnosis in pregnancy trimester 2** | |
|  |  |
|  |  |

**Figure S8 continued.** Forest plots showing the pooled prevalence of antimycotics in the 30 days pre-COVID (left) and 30 days post-COVID (right) in non-hospitalized pregnant women with COVID-19 (upper) and pregnant women without COVID-19 (lower), by pregnancy trimester

| **COVID-19 diagnosis in pregnancy trimester 3** | |
| --- | --- |
|  |  |
|  |  |

# **Figure S9.** Forest plots showing the pooled prevalence of antineoplastic agents in the 30 days pre-COVID (left) and 30 days post-COVID (right) in non-hospitalized pregnant women with COVID-19 (upper) and pregnant women without COVID-19 (lower), by pregnancy trimester

| **COVID-19 diagnosis in pregnancy trimester 1** | |
| --- | --- |
|  |  |
|  |  |
| **COVID-19 diagnosis in pregnancy trimester 2** | |
|  |  |
|  |  |

**Figure S9 continued.** Forest plots showing the pooled prevalence of antineoplastic agents in the 30 days pre-COVID (left) and 30 days post-COVID (right) in non-hospitalized pregnant women with COVID-19 (upper) and pregnant women without COVID-19 (lower), by pregnancy trimester

| **COVID-19 diagnosis in pregnancy trimester 3** | |
| --- | --- |
|  |  |
|  |  |

# **Figure S10.** Forest plots showing the pooled prevalence of antiprotozoals in the 30 days pre-COVID (left) and 30 days post-COVID (right) in non-hospitalized pregnant women with COVID-19 (upper) and pregnant women without COVID-19 (lower), by pregnancy trimester

| **COVID-19 diagnosis in pregnancy trimester 1** | |
| --- | --- |
|  |  |
|  |  |
| **COVID-19 diagnosis in pregnancy trimester 2** | |
|  |  |
|  |  |

**Figure S10 continued.** Forest plots showing the pooled prevalence of antiprotozoals in the 30 days pre-COVID (left) and 30 days post-COVID (right) in non-hospitalized pregnant women with COVID-19 (upper) and pregnant women without COVID-19 (lower), by pregnancy trimester

| **COVID-19 diagnosis in pregnancy trimester 3** | |
| --- | --- |
|  |  |
|  |  |

# **Figure S11.** Forest plots showing the pooled prevalence of antithrombotic agents in the 30 days pre-COVID (left) and 30 days post-COVID (right) in non-hospitalized pregnant women with COVID-19 (upper) and pregnant women without COVID-19 (lower), by pregnancy trimester

| **COVID-19 diagnosis in pregnancy trimester 1** | |
| --- | --- |
|  |  |
|  |  |
| **COVID-19 diagnosis in pregnancy trimester 2** | |
|  |  |
|  |  |

**Figure S11 continued.** Forest plots showing the pooled prevalence of antithrombotic agents in the 30 days pre-COVID (left) and 30 days post-COVID (right) in non-hospitalized pregnant women with COVID-19 (upper) and pregnant women without COVID-19 (lower), by pregnancy trimester

| **COVID-19 diagnosis in pregnancy trimester 3** | |
| --- | --- |
|  |  |
|  |  |

# **Figure S12.** Forest plots showing the pooled prevalence of antivirals in the 30 days pre-COVID (left) and 30 days post-COVID (right) in non-hospitalized pregnant women with COVID-19 (upper) and pregnant women without COVID-19 (lower), by pregnancy trimester

| **COVID-19 diagnosis in pregnancy trimester 1** | |
| --- | --- |
|  |  |
|  |  |
| **COVID-19 diagnosis in pregnancy trimester 2** | |
|  |  |
|  |  |

**Figure S12 continued.** Forest plots showing the pooled prevalence of antivirals in the 30 days pre-COVID (left) and 30 days post-COVID (right) in non-hospitalized pregnant women with COVID-19 (upper) and pregnant women without COVID-19 (lower), by pregnancy trimester

| **COVID-19 diagnosis in pregnancy trimester 3** | |
| --- | --- |
|  |  |
|  |  |

# **Figure S13.** Forest plots showing the pooled prevalence of corticosteroids in the 30 days pre-COVID (left) and 30 days post-COVID (right) in non-hospitalized pregnant women with COVID-19 (upper) and pregnant women without COVID-19 (lower), by pregnancy trimester

| **COVID-19 diagnosis in pregnancy trimester 1** | |
| --- | --- |
|  |  |
|  |  |
| **COVID-19 diagnosis in pregnancy trimester 2** | |
|  |  |
|  |  |

**Figure S13 continued.** Forest plots showing the pooled prevalence of corticosteroids in the 30 days pre-COVID (left) and 30 days post-COVID (right) in non-hospitalized pregnant women with COVID-19 (upper) and pregnant women without COVID-19 (lower), by pregnancy trimester

| **COVID-19 diagnosis in pregnancy trimester 3** | |
| --- | --- |
|  |  |
|  |  |

# **Figure S14.** Forest plots showing the pooled prevalence of cough and cold preparations in the 30 days pre-COVID (left) and 30 days post-COVID (right) in non-hospitalized pregnant women with COVID-19 (upper) and pregnant women without COVID-19 (lower), by pregnancy trimester

| **COVID-19 diagnosis in pregnancy trimester 1** | |
| --- | --- |
|  |  |
|  |  |
| **COVID-19 diagnosis in pregnancy trimester 2** | |
|  |  |
|  |  |

**Figure S14 continued.** Forest plots showing the pooled prevalence of cough and cold preparations in the 30 days pre-COVID (left) and 30 days post-COVID (right) in non-hospitalized pregnant women with COVID-19 (upper) and pregnant women without COVID-19 (lower), by pregnancy trimester

| **COVID-19 diagnosis in pregnancy trimester 3** | |
| --- | --- |
|  |  |
|  |  |

# **Figure S15.** Forest plots showing the pooled prevalence of drugs for obstructive airway diseases in the 30 days pre-COVID (left) and 30 days post-COVID (right) in non-hospitalized pregnant women with COVID-19 (upper) and pregnant women without COVID-19 (lower), by pregnancy trimester

| **COVID-19 diagnosis in pregnancy trimester 1** | |
| --- | --- |
|  |  |
|  |  |
| **COVID-19 diagnosis in pregnancy trimester 2** | |
|  |  |
|  |  |

**Figure S15 continued.** Forest plots showing the pooled prevalence of drugs for obstructive airway diseases in the 30 days pre-COVID (left) and 30 days post-COVID (right) in non-hospitalized pregnant women with COVID-19 (upper) and pregnant women without COVID-19 (lower), by pregnancy trimester

| **COVID-19 diagnosis in pregnancy trimester 3** | |
| --- | --- |
|  |  |
|  |  |

# **Figure S16.** Forest plots showing the pooled prevalence of drugs used in diabetes in the 30 days pre-COVID (left) and 30 days post-COVID (right) in non-hospitalized pregnant women with COVID-19 (upper) and pregnant women without COVID-19 (lower), by pregnancy trimester

| **COVID-19 diagnosis in pregnancy trimester 1** | |
| --- | --- |
|  |  |
|  |  |
| **COVID-19 diagnosis in pregnancy trimester 2** | |
|  |  |
|  |  |

**Figure S16 continued.** Forest plots showing the pooled prevalence of drugs used in diabetes in the 30 days pre-COVID (left) and 30 days post-COVID (right) in non-hospitalized pregnant women with COVID-19 (upper) and pregnant women without COVID-19 (lower), by pregnancy trimester

| **COVID-19 diagnosis in pregnancy trimester 3** | |
| --- | --- |
|  |  |
|  |  |

# **Figure S17.** Forest plots showing the pooled prevalence of immune sera and immunoglobulins in the 30 days pre-COVID (left) and 30 days post-COVID (right) in non-hospitalized pregnant women with COVID-19 (upper) and pregnant women without COVID-19 (lower), by pregnancy trimester

| **COVID-19 diagnosis in pregnancy trimester 1** | |
| --- | --- |
|  |  |
|  |  |
| **COVID-19 diagnosis in pregnancy trimester 2** | |
|  |  |
|  |  |

**Figure S17 continued.** Forest plots showing the pooled prevalence of immune sera and immunoglobulins in the 30 days pre-COVID (left) and 30 days post-COVID (right) in non-hospitalized pregnant women with COVID-19 (upper) and pregnant women without COVID-19 (lower), by pregnancy trimester

| **COVID-19 diagnosis in pregnancy trimester 3** | |
| --- | --- |
|  |  |
|  |  |

# **Figure S18.** Forest plots showing the pooled prevalence of immunostimulants in the 30 days pre-COVID (left) and 30 days post-COVID (right) in non-hospitalized pregnant women with COVID-19 (upper) and pregnant women without COVID-19 (lower), by pregnancy trimester

| **COVID-19 diagnosis in pregnancy trimester 1** | |
| --- | --- |
|  |  |
|  |  |
| **COVID-19 diagnosis in pregnancy trimester 2** | |
|  |  |
|  |  |

**Figure S18 continued.** Forest plots showing the pooled prevalence of immunostimulants in the 30 days pre-COVID (left) and 30 days post-COVID (right) in non-hospitalized pregnant women with COVID-19 (upper) and pregnant women without COVID-19 (lower), by pregnancy trimester

| **COVID-19 diagnosis in pregnancy trimester 3** | |
| --- | --- |
|  |  |
|  |  |

# **Figure S19.** Forest plots showing the pooled prevalence of immunosuppressants in the 30 days pre-COVID (left) and 30 days post-COVID (right) in non-hospitalized pregnant women with COVID-19 (upper) and pregnant women without COVID-19 (lower), by pregnancy trimester

| **COVID-19 diagnosis in pregnancy trimester 1** | |
| --- | --- |
|  |  |
|  |  |
| **COVID-19 diagnosis in pregnancy trimester 2** | |
|  |  |
|  |  |

**Figure S19 continued.** Forest plots showing the pooled prevalence of immunosuppressants in the 30 days pre-COVID (left) and 30 days post-COVID (right) in non-hospitalized pregnant women with COVID-19 (upper) and pregnant women without COVID-19 (lower), by pregnancy trimester

| **COVID-19 diagnosis in pregnancy trimester 3** | |
| --- | --- |
|  |  |
|  |  |

# **Figure S20.** Forest plots showing the pooled prevalence of nasal preparations in the 30 days pre-COVID (left) and 30 days post-COVID (right) in non-hospitalized pregnant women with COVID-19 (upper) and pregnant women without COVID-19 (lower), by pregnancy trimester

| **COVID-19 diagnosis in pregnancy trimester 1** | |
| --- | --- |
|  |  |
|  |  |
| **COVID-19 diagnosis in pregnancy trimester 2** | |
|  |  |
|  |  |

**Figure S20 continued.** Forest plots showing the pooled prevalence of nasal preparations in the 30 days pre-COVID (left) and 30 days post-COVID (right) in non-hospitalized pregnant women with COVID-19 (upper) and pregnant women without COVID-19 (lower), by pregnancy trimester

| **COVID-19 diagnosis in pregnancy trimester 3** | |
| --- | --- |
|  |  |
|  |  |

# **Figure S21.** Forest plots showing the pooled prevalence of psychoanaleptics in the 30 days pre-COVID (left) and 30 days post-COVID (right) in non-hospitalized pregnant women with COVID-19 (upper) and pregnant women without COVID-19 (lower), by pregnancy trimester

| **COVID-19 diagnosis in pregnancy trimester 1** | |
| --- | --- |
|  |  |
|  |  |
| **COVID-19 diagnosis in pregnancy trimester 2** | |
|  |  |
|  |  |

**Figure S21 continued.** Forest plots showing the pooled prevalence of psychoanaleptics in the 30 days pre-COVID (left) and 30 days post-COVID (right) in non-hospitalized pregnant women with COVID-19 (upper) and pregnant women without COVID-19 (lower), by pregnancy trimester

| **COVID-19 diagnosis in pregnancy trimester 3** | |
| --- | --- |
|  |  |
|  |  |

# **Figure S22.** Forest plots showing the pooled prevalence of psycholeptics in the 30 days pre-COVID (left) and 30 days post-COVID (right) in non-hospitalized pregnant women with COVID-19 (upper) and pregnant women without COVID-19 (lower), by pregnancy trimester

| **COVID-19 diagnosis in pregnancy trimester 1** | |
| --- | --- |
|  |  |
|  |  |
| **COVID-19 diagnosis in pregnancy trimester 2** | |
|  |  |
|  |  |

**Figure S22 continued.** Forest plots showing the pooled prevalence of psycholeptics in the 30 days pre-COVID (left) and 30 days post-COVID (right) in non-hospitalized pregnant women with COVID-19 (upper) and pregnant women without COVID-19 (lower), by pregnancy trimester

| **COVID-19 diagnosis in pregnancy trimester 3** | |
| --- | --- |
|  |  |
|  |  |

# **Table S7.** Baseline characteristics of pregnant women with COVID-19 and non-pregnant women with COVID-19

|  | **Trimester at  COVID-19 infection** | **Age** | | **Co-morbidities^1^** | |
| --- | --- | --- | --- | --- | --- |
|  | **Pregnant with**  **COVID-19** | **Pregnant with**  **COVID-19** | **Non-pregnant with  COVID-19** | **Pregnant with  COVID-19** | **Non-pregnant with  COVID-19** |
| Tuscany,  Italy | 1^st^ trimester: 24.5%  2^nd^ trimester: 35.3%  3^rd^ trimester: 40.2% | 12-24 years: 10.7%  25-39 years: 82.5%  40-55 years: 6.8% | 12-24 years: 11.2%  25-39 years: 81.7%  40-55 years: 7.0% | Any: 21.3% Cardiovascular: 4.1%  Chronic lung: 4.7%  Severe obesity: 0.3% | Any: 24.5% Cardiovascular: 3.9%  Chronic lung: 6.4%  Severe obesity: 0.1% |
| Valencia, Spain | 1^st^ trimester: 38.4%  2^nd^ trimester: 31.9%  3^rd^ trimester: 29.7% | 12-24 years: 12.5%  25-39 years: 77.3%  40-55 years: 10.2% | 12-24 years: 13.2%  25-39 years: 76.9%  40-55 years: 9.9% | Any: 24.2% Cardiovascular: 6.2%  Chronic lung: 10.1%  Severe obesity: 1.8% | Any: 23.7% Cardiovascular: 4.5%  Chronic lung: 10.2%  Severe obesity: 1.9% |
| Aragon,  Spain | 1^st^ trimester: 26.7%  2^nd^ trimester: 36.1%  3^rd^ trimester: 37.3% | 12-24 years: 13.0%  25-39 years: 78.6%  40-55 years: 8.4% | 12-24 years: 13.8%  25-39 years: 78.2%  40-55 years: 8.0% | Any: 25.6% Cardiovascular: 4.6%  Chronic lung: 8.2%  Severe obesity: 0.4% | Any: 26.6% Cardiovascular: 3.0%  Chronic lung: 8.5%  Severe obesity: 1.1% |
| Norway | 1^st^ trimester: 24.7%  2^nd^ trimester: 41.1%  3^rd^ trimester: 34.2% | 12-24 years: 10.4%  25-39 years: 87.1%  40-55 years: 2.5% | 12-24 years: 10.6%  25-39 years: 86.1%  40-55 years: 3.3% | Any: 18.5% Cardiovascular: 3.5%  Chronic lung: 4.3%  Severe obesity: 1.5% | Any: 20.9% Cardiovascular: 1.9%  Chronic lung: 6.1%  Severe obesity: 1.3% |
| Sweden | 1^st^ trimester: 6.1%  2^nd^ trimester: 34.7%  3^rd^ trimester: 59.2% | 12-24 years: 6.1%  25-39 years: 88.6%  40-55 years: 5.3% | 12-24 years: 6.1%  25-39 years: 88.6%  40-55 years: 5.3% | Any: 5.8% Cardiovascular: 0.4%  Chronic lung: 0.8%  Severe obesity: 3.4% | Any: 16.3% Cardiovascular: 1.7%  Chronic lung: 6.2%  Severe obesity: 7.0% |
| Alberta, Canada | 1^st^ trimester: 29.0%  2^nd^ trimester: 35.9%  3^rd^ trimester: 35.1% | 15-24 years: 14.8%  25-39 years: 81.9%  40-45 years: 3.3% | 15-24 years: 31.5%  25-39 years: 50.2%  40-45 years: 19.7% | Any: 41.4% Cardiovascular: 2.6%  Respiratory: 7.1%  Severe obesity: 1.8% | Any: 47.3% Cardiovascular: 2.3%  Respiratory: 8.8%  Severe obesity: 2.0% |
| Manitoba, Canada | 1^st^ trimester: 11.2%  2^nd^ trimester: 36.5%  3^rd^ trimester: 52.4% | 15-24 years: >3.5%  25-39 years: 70.6%  40-45 years: <3.5% | 15-24 years: 32.7%  25-39 years: 48.8%  40-45 years: 18.6% | Any: 36.5% Cardiovascular: <3.5%  Respiratory: >3.5%  Severe obesity: <3.5% | Any: 42.1% Cardiovascular: 1.4%  Respiratory: 7.7%  Severe obesity: 1.6% |
| Ontario, Canada | 1^st^ trimester: 28.9%  2^nd^ trimester: 32.5%  3^rd^ trimester: 38.6% | 15-24 years: 34.7%  25-39 years: 61.7%  40-45 years: 3.5% | 15-24 years: 44.1%  25-39 years: 37.6%  40-45 years: 18.3% | Any: 28.9% Cardiovascular: 0%  Respiratory: 2.6%  Severe obesity: 1.2% | Any: 26.6% Cardiovascular: 0.1%  Respiratory: 2.9%  Severe obesity: 2.5% |
| U.S. | 1^st^ trimester: 19.7%  2^nd^ trimester: 25.7%  3^rd^ trimester: 54.5% | 12-24 years: NA  25-39 years: NA  40-55 years: NA | 12-24 years: NA  25-39 years: NA  40-55 years: NA | Any: NA Cardiovascular: 0.9%  Chronic lung: 4.3%  Severe obesity: 14.3% | Any comorbidities: NA Cardiovascular: 1.0%  Chronic lung: 4.9%  Severe obesity: 12.7% |

^1^Any co-morbidities included: cancer, cardiovascular disease, chronic kidney disease, chronic liver disease, chronic lung disease, common rheumatic disease, diabetes, HIV, hypertension, mental disorders, severe obesity, sickle cell disease, and use of immunosuppressants. Abbreviations: N.A. = not available.

# **Figure S23.** Forest plots showing the pooled prevalence of analgesics in the 30 days pre-COVID (left) and 30 days post-COVID (right) in non-hospitalized pregnant women with COVID-19 (upper) and non-hospitalized non-pregnant women with COVID-19 (lower), by pregnancy trimester

| **COVID-19 diagnosis in pregnancy trimester 1** | |
| --- | --- |
|  |  |
|  |  |
| **COVID-19 diagnosis in pregnancy trimester 2** | |
|  |  |
|  |  |

**Figure S23 continued.** Forest plots showing the pooled prevalence of analgesics in the 30 days pre-COVID (left) and 30 days post-COVID (right) in non-hospitalized pregnant women with COVID-19 (upper) and non-hospitalized non-pregnant women with COVID-19 (lower), by pregnancy trimester

| **COVID-19 diagnosis in pregnancy trimester 3** | |
| --- | --- |
|  |  |
|  |  |

# **Figure S24.** Forest plots showing the pooled prevalence of anthelminthics in the 30 days pre-COVID (left) and 30 days post-COVID (right) in non-hospitalized pregnant women with COVID-19 (upper) and non-hospitalized non-pregnant women with COVID-19 (lower), by pregnancy trimester

| **COVID-19 diagnosis in pregnancy trimester 1** | |
| --- | --- |
|  |  |
|  |  |
| **COVID-19 diagnosis in pregnancy trimester 2** | |
|  |  |
|  |  |

**Figure S24 continued.** Forest plots showing the pooled prevalence of anthelminthics in the 30 days pre-COVID (left) and 30 days post-COVID (right) in non-hospitalized pregnant women with COVID-19 (upper) and non-hospitalized non-pregnant women with COVID-19 (lower), by pregnancy trimester

| **COVID-19 diagnosis in pregnancy trimester 3** | |
| --- | --- |
|  |  |
|  |  |

# **Figure S25.** Forest plots showing the pooled prevalence of anti-inflammatory and antirheumatic products in the 30 days pre-COVID (left) and 30 days post-COVID (right) in non-hospitalized pregnant women with COVID-19 (upper) and non-hospitalized non-pregnant women with COVID-19 (lower), by pregnancy trimester

| **COVID-19 diagnosis in pregnancy trimester 1** | |
| --- | --- |
|  |  |
|  |  |
| **COVID-19 diagnosis in pregnancy trimester 2** | |
|  |  |
|  |  |

**Figure S25 continued.** Forest plots showing the pooled prevalence of anti-inflammatory and antirheumatic products in the 30 days pre-COVID (left) and 30 days post-COVID (right) in non-hospitalized pregnant women with COVID-19 (upper) and non-hospitalized non-pregnant women with COVID-19 (lower), by pregnancy trimester

| **COVID-19 diagnosis in pregnancy trimester 3** | |
| --- | --- |
|  |  |
|  |  |

# **Figure S26.** Forest plots showing the pooled prevalence of antibacterials in the 30 days pre-COVID (left) and 30 days post-COVID (right) in non-hospitalized pregnant women with COVID-19 (upper) and non-hospitalized non-pregnant women with COVID-19 (lower), by pregnancy trimester

| **COVID-19 diagnosis in pregnancy trimester 1** | |
| --- | --- |
|  |  |
|  |  |
| **COVID-19 diagnosis in pregnancy trimester 2** | |
|  |  |
|  |  |

**Figure S26 continued.** Forest plots showing the pooled prevalence of antibacterials in the 30 days pre-COVID (left) and 30 days post-COVID (right) in non-hospitalized pregnant women with COVID-19 (upper) and non-hospitalized non-pregnant women with COVID-19 (lower), by pregnancy trimester

| **COVID-19 diagnosis in pregnancy trimester 3** | |
| --- | --- |
|  |  |
|  |  |

# **Figure S27.** Forest plots showing the pooled prevalence of antigout preparations in the 30 days pre-COVID (left) and 30 days post-COVID (right) in non-hospitalized pregnant women with COVID-19 (upper) and non-hospitalized non-pregnant women with COVID-19 (lower), by pregnancy trimester

| **COVID-19 diagnosis in pregnancy trimester 1** | |
| --- | --- |
|  |  |
|  |  |
| **COVID-19 diagnosis in pregnancy trimester 2** | |
|  |  |
|  |  |

**Figure S27 continued.** Forest plots showing the pooled prevalence of antigout preparations in the 30 days pre-COVID (left) and 30 days post-COVID (right) in non-hospitalized pregnant women with COVID-19 (upper) and non-hospitalized non-pregnant women with COVID-19 (lower), by pregnancy trimester

| **COVID-19 diagnosis in pregnancy trimester 3** | |
| --- | --- |
|  |  |
|  |  |

# **Figure S28.** Forest plots showing the pooled prevalence of antihypertensives in the 30 days pre-COVID (left) and 30 days post-COVID (right) in non-hospitalized pregnant women with COVID-19 (upper) and non-hospitalized non-pregnant women with COVID-19 (lower), by pregnancy trimester

| **COVID-19 diagnosis in pregnancy trimester 1** | |
| --- | --- |
|  |  |
|  |  |
| **COVID-19 diagnosis in pregnancy trimester 2** | |
|  |  |
|  |  |

**Figure S28 continued.** Forest plots showing the pooled prevalence of antihypertensives in the 30 days pre-COVID (left) and 30 days post-COVID (right) in non-hospitalized pregnant women with COVID-19 (upper) and non-hospitalized non-pregnant women with COVID-19 (lower), by pregnancy trimester

| **COVID-19 diagnosis in pregnancy trimester 3** | |
| --- | --- |
|  |  |
|  |  |

# **Figure S29.** Forest plots showing the pooled prevalence of antimycobacterials in the 30 days pre-COVID (left) and 30 days post-COVID (right) in non-hospitalized pregnant women with COVID-19 (upper) and non-hospitalized non-pregnant women with COVID-19 (lower), by pregnancy trimester

| **COVID-19 diagnosis in pregnancy trimester 1** | |
| --- | --- |
|  |  |
|  |  |
| **COVID-19 diagnosis in pregnancy trimester 2** | |
|  |  |
|  |  |

**Figure S29 continued.** Forest plots showing the pooled prevalence of antimycobacterials in the 30 days pre-COVID (left) and 30 days post-COVID (right) in non-hospitalized pregnant women with COVID-19 (upper) and non-hospitalized non-pregnant women with COVID-19 (lower), by pregnancy trimester

| **COVID-19 diagnosis in pregnancy trimester 3** | |
| --- | --- |
|  |  |
|  |  |

# **Figure S30.** Forest plots showing the pooled prevalence of antimycotics in the 30 days pre-COVID (left) and 30 days post-COVID (right) in non-hospitalized pregnant women with COVID-19 (upper) and non-hospitalized non-pregnant women with COVID-19 (lower), by pregnancy trimester

| **COVID-19 diagnosis in pregnancy trimester 1** | |
| --- | --- |
|  |  |
|  |  |
| **COVID-19 diagnosis in pregnancy trimester 2** | |
|  |  |
|  |  |

**Figure S30 continued.** Forest plots showing the pooled prevalence of antimycotics in the 30 days pre-COVID (left) and 30 days post-COVID (right) in non-hospitalized pregnant women with COVID-19 (upper) and non-hospitalized non-pregnant women with COVID-19 (lower), by pregnancy trimester

| **COVID-19 diagnosis in pregnancy trimester 3** | |
| --- | --- |
|  |  |
|  |  |

# **Figure S31.** Forest plots showing the pooled prevalence of antineoplastic agents in the 30 days pre-COVID (left) and 30 days post-COVID (right) in non-hospitalized pregnant women with COVID-19 (upper) and non-hospitalized non-pregnant women with COVID-19 (lower), by pregnancy trimester

| **COVID-19 diagnosis in pregnancy trimester 1** | |
| --- | --- |
|  |  |
|  |  |
| **COVID-19 diagnosis in pregnancy trimester 2** | |
|  |  |
|  |  |

**Figure S31 continued.** Forest plots showing the pooled prevalence of antineoplastic agents in the 30 days pre-COVID (left) and 30 days post-COVID (right) in non-hospitalized pregnant women with COVID-19 (upper) and non-hospitalized non-pregnant women with COVID-19 (lower), by pregnancy trimester

| **COVID-19 diagnosis in pregnancy trimester 3** | |
| --- | --- |
|  |  |
|  |  |

# **Figure S32.** Forest plots showing the pooled prevalence of antiprotozoals in the 30 days pre-COVID (left) and 30 days post-COVID (right) in non-hospitalized pregnant women with COVID-19 (upper) and non-hospitalized non-pregnant women with COVID-19 (lower), by pregnancy trimester

| **COVID-19 diagnosis in pregnancy trimester 1** | |
| --- | --- |
|  |  |
|  |  |
| **COVID-19 diagnosis in pregnancy trimester 2** | |
|  |  |
|  |  |

**Figure S32 continued.** Forest plots showing the pooled prevalence of antiprotozoals in the 30 days pre-COVID (left) and 30 days post-COVID (right) in non-hospitalized pregnant women with COVID-19 (upper) and non-hospitalized non-pregnant women with COVID-19 (lower), by pregnancy trimester

| **COVID-19 diagnosis in pregnancy trimester 3** | |
| --- | --- |
|  |  |
|  |  |

# **Figure S33.** Forest plots showing the pooled prevalence of antithrombotic agents in the 30 days pre-COVID (left) and 30 days post-COVID (right) in non-hospitalized pregnant women with COVID-19 (upper) and non-hospitalized non-pregnant women with COVID-19 (lower), by pregnancy trimester

| **COVID-19 diagnosis in pregnancy trimester 1** | |
| --- | --- |
|  |  |
|  |  |
| **COVID-19 diagnosis in pregnancy trimester 2** | |
|  |  |
|  |  |

**Figure S33 continued.** Forest plots showing the pooled prevalence of antithrombotic agents in the 30 days pre-COVID (left) and 30 days post-COVID (right) in non-hospitalized pregnant women with COVID-19 (upper) and non-hospitalized non-pregnant women with COVID-19 (lower), by pregnancy trimester

| **COVID-19 diagnosis in pregnancy trimester 3** | |
| --- | --- |
|  |  |
|  |  |

# **Figure S34.** Forest plots showing the pooled prevalence of antivirals in the 30 days pre-COVID (left) and 30 days post-COVID (right) in non-hospitalized pregnant women with COVID-19 (upper) and non-hospitalized non-pregnant women with COVID-19 (lower), by pregnancy trimester

| **COVID-19 diagnosis in pregnancy trimester 1** | |
| --- | --- |
|  |  |
|  |  |
| **COVID-19 diagnosis in pregnancy trimester 2** | |
|  |  |
|  |  |

**Figure S34 continued.** Forest plots showing the pooled prevalence of antivirals in the 30 days pre-COVID (left) and 30 days post-COVID (right) in non-hospitalized pregnant women with COVID-19 (upper) and non-hospitalized non-pregnant women with COVID-19 (lower), by pregnancy trimester

| **COVID-19 diagnosis in pregnancy trimester 3** | |
| --- | --- |
|  |  |
|  |  |

# **Figure S35.** Forest plots showing the pooled prevalence of corticosteroids in the 30 days pre-COVID (left) and 30 days post-COVID (right) in non-hospitalized pregnant women with COVID-19 (upper) and non-hospitalized non-pregnant women with COVID-19 (lower), by pregnancy trimester

| **COVID-19 diagnosis in pregnancy trimester 1** | |
| --- | --- |
|  |  |
|  |  |
| **COVID-19 diagnosis in pregnancy trimester 2** | |
|  |  |
|  |  |

**Figure S35 continued.** Forest plots showing the pooled prevalence of corticosteroids in the 30 days pre-COVID (left) and 30 days post-COVID (right) in non-hospitalized pregnant women with COVID-19 (upper) and non-hospitalized non-pregnant women with COVID-19 (lower), by pregnancy trimester

| **COVID-19 diagnosis in pregnancy trimester 3** | |
| --- | --- |
|  |  |
|  |  |

# **Figure S36.** Forest plots showing the pooled prevalence of cough and cold preparations in the 30 days pre-COVID (left) and 30 days post-COVID (right) in non-hospitalized pregnant women with COVID-19 (upper) and non-hospitalized non-pregnant women with COVID-19 (lower), by pregnancy trimester

| **COVID-19 diagnosis in pregnancy trimester 1** | |
| --- | --- |
|  |  |
|  |  |
| **COVID-19 diagnosis in pregnancy trimester 2** | |
|  |  |
|  |  |

**Figure S36 continued.** Forest plots showing the pooled prevalence of cough and cold preparations in the 30 days pre-COVID (left) and 30 days post-COVID (right) in non-hospitalized pregnant women with COVID-19 (upper) and non-hospitalized non-pregnant women with COVID-19 (lower), by pregnancy trimester

| **COVID-19 diagnosis in pregnancy trimester 3** | |
| --- | --- |
|  |  |
|  |  |

# **Figure S37.** Forest plots showing the pooled prevalence of drugs for obstructive airway diseases in the 30 days pre-COVID (left) and 30 days post-COVID (right) in non-hospitalized pregnant women with COVID-19 (upper) and non-hospitalized non-pregnant women with COVID-19 (lower), by pregnancy trimester

| **COVID-19 diagnosis in pregnancy trimester 1** | |
| --- | --- |
|  |  |
|  |  |
| **COVID-19 diagnosis in pregnancy trimester 2** | |
|  |  |
|  |  |

**Figure S37 continued.** Forest plots showing the pooled prevalence of drugs for obstructive airway diseases in the 30 days pre-COVID (left) and 30 days post-COVID (right) in non-hospitalized pregnant women with COVID-19 (upper) and non-hospitalized non-pregnant women with COVID-19 (lower), by pregnancy trimester

| **COVID-19 diagnosis in pregnancy trimester 3** | |
| --- | --- |
|  |  |
|  |  |

# **Figure S38.** Forest plots showing the pooled prevalence of drugs used in diabetes in the 30 days pre-COVID (left) and 30 days post-COVID (right) in non-hospitalized pregnant women with COVID-19 (upper) and non-hospitalized non-pregnant women with COVID-19 (lower), by pregnancy trimester

| **COVID-19 diagnosis in pregnancy trimester 1** | |
| --- | --- |
|  |  |
|  |  |
| **COVID-19 diagnosis in pregnancy trimester 2** | |
|  |  |
|  |  |

**Figure S38 continued.** Forest plots showing the pooled prevalence of drugs used in diabetes in the 30 days pre-COVID (left) and 30 days post-COVID (right) in non-hospitalized pregnant women with COVID-19 (upper) and non-hospitalized non-pregnant women with COVID-19 (lower), by pregnancy trimester

| **COVID-19 diagnosis in pregnancy trimester 3** | |
| --- | --- |
|  |  |
|  |  |

# **Figure S39.** Forest plots showing the pooled prevalence of immune sera and immunoglobulins in the 30 days pre-COVID (left) and 30 days post-COVID (right) in non-hospitalized pregnant women with COVID-19 (upper) and non-hospitalized non-pregnant women with COVID-19 (lower), by pregnancy trimester

| **COVID-19 diagnosis in pregnancy trimester 1** | |
| --- | --- |
|  |  |
|  |  |
| **COVID-19 diagnosis in pregnancy trimester 2** | |
|  |  |
|  |  |

**Figure S39 continued.** Forest plots showing the pooled prevalence of immune sera and immunoglobulins in the 30 days pre-COVID (left) and 30 days post-COVID (right) in non-hospitalized pregnant women with COVID-19 (upper) and non-hospitalized non-pregnant women with COVID-19 (lower), by pregnancy trimester

| **COVID-19 diagnosis in pregnancy trimester 3** | |
| --- | --- |
|  |  |
|  |  |

# **Figure S40.** Forest plots showing the pooled prevalence of immunostimulants in the 30 days pre-COVID (left) and 30 days post-COVID (right) in non-hospitalized pregnant women with COVID-19 (upper) and non-hospitalized non-pregnant women with COVID-19 (lower), by pregnancy trimester

| **COVID-19 diagnosis in pregnancy trimester 1** | |
| --- | --- |
|  |  |
|  |  |
| **COVID-19 diagnosis in pregnancy trimester 2** | |
|  |  |
|  |  |

**Figure S40 continued.** Forest plots showing the pooled prevalence of immunostimulants in the 30 days pre-COVID (left) and 30 days post-COVID (right) in non-hospitalized pregnant women with COVID-19 (upper) and non-hospitalized non-pregnant women with COVID-19 (lower), by pregnancy trimester

| **COVID-19 diagnosis in pregnancy trimester 3** | |
| --- | --- |
|  |  |
|  |  |

# **Figure S41.** Forest plots showing the pooled prevalence of immunosuppressants in the 30 days pre-COVID (left) and 30 days post-COVID (right) in non-hospitalized pregnant women with COVID-19 (upper) and non-hospitalized non-pregnant women with COVID-19 (lower), by pregnancy trimester

| **COVID-19 diagnosis in pregnancy trimester 1** | |
| --- | --- |
|  |  |
|  |  |
| **COVID-19 diagnosis in pregnancy trimester 2** | |
|  |  |
|  |  |

**Figure S41 continued.** Forest plots showing the pooled prevalence of immunosuppressants in the 30 days pre-COVID (left) and 30 days post-COVID (right) in non-hospitalized pregnant women with COVID-19 (upper) and non-hospitalized non-pregnant women with COVID-19 (lower), by pregnancy trimester

| **COVID-19 diagnosis in pregnancy trimester 3** | |
| --- | --- |
|  |  |
|  |  |

# **Figure S42.** Forest plots showing the pooled prevalence of nasal preparations in the 30 days pre-COVID (left) and 30 days post-COVID (right) in non-hospitalized pregnant women with COVID-19 (upper) and non-hospitalized non-pregnant women with COVID-19 (lower), by pregnancy trimester

| **COVID-19 diagnosis in pregnancy trimester 1** | |
| --- | --- |
|  |  |
|  |  |
| **COVID-19 diagnosis in pregnancy trimester 2** | |
|  |  |
|  |  |

**Figure S42 continued.** Forest plots showing the pooled prevalence of nasal preparations in the 30 days pre-COVID (left) and 30 days post-COVID (right) in non-hospitalized pregnant women with COVID-19 (upper) and non-hospitalized non-pregnant women with COVID-19 (lower), by pregnancy trimester

| **COVID-19 diagnosis in pregnancy trimester 3** | |
| --- | --- |
|  |  |
|  |  |

# **Figure S43.** Forest plots showing the pooled prevalence of psychoanaleptics in the 30 days pre-COVID (left) and 30 days post-COVID (right) in non-hospitalized pregnant women with COVID-19 (upper) and non-hospitalized non-pregnant women with COVID-19 (lower), by pregnancy trimester

| **COVID-19 diagnosis in pregnancy trimester 1** | |
| --- | --- |
|  |  |
|  |  |
| **COVID-19 diagnosis in pregnancy trimester 2** | |
|  |  |
|  |  |

**Figure S43 continued.** Forest plots showing the pooled prevalence of psychoanaleptics in the 30 days pre-COVID (left) and 30 days post-COVID (right) in non-hospitalized pregnant women with COVID-19 (upper) and non-hospitalized non-pregnant women with COVID-19 (lower), by pregnancy trimester

| **COVID-19 diagnosis in pregnancy trimester 3** | |
| --- | --- |
|  |  |
|  |  |

# **Figure S44.** Forest plots showing the pooled prevalence of psycholeptics in the 30 days pre-COVID (left) and 30 days post-COVID (right) in non-hospitalized pregnant women with COVID-19 (upper) and non-hospitalized non-pregnant women with COVID-19 (lower), by pregnancy trimester

| **COVID-19 diagnosis in pregnancy trimester 1** | |
| --- | --- |
|  |  |
|  |  |
| **COVID-19 diagnosis in pregnancy trimester 2** | |
|  |  |
|  |  |

**Figure S44 continued.** Forest plots showing the pooled prevalence of psycholeptics in the 30 days pre-COVID (left) and 30 days post-COVID (right) in non-hospitalized pregnant women with COVID-19 (upper) and non-hospitalized non-pregnant women with COVID-19 (lower), by pregnancy trimester

| **COVID-19 diagnosis in pregnancy trimester 3** | |
| --- | --- |
|  |  |
|  |  |

# **Table S8.** Sensitivity analysis: effects of excluding Canadian cohorts on pooled prevalence estimates in non-hospitalized pregnant women with COVID-19 versus those without COVID-19

|  |  | **Non-hospitalized pregnant women**  **with COVID-19** | | | | **Pregnant women**  **without COVID-19** | | | |
| --- | --- | --- | --- | --- | --- | --- | --- | --- | --- |
|  | *Trim* | *Original analysis* | *I^2^* | *Sensitivity analysis* | *I^2^* | *Original analysis* | *I^2^* | *Sensitivity analysis* | *I^2^* |
| Analgesics | 1 | 2,8 (1,7 - 4,4) | 76 | 2,7 (1,4 - 5,2) | 83 | 2,0 (1,2 - 3,3) | 99 | 1,4 (0,8 - 2,6) | 77 |
|  | 2 | 1,8 (0,9 - 3,8) | 87 | 2,5 (1,2 - 4,9) | 89 | 1,6 (1,0 - 2,8) | 97 | 1,2 (0,6 - 2,2) | 85 |
|  | 3 | 3,4 (1,6 - 6,8) | 98 | 3,3 (1,2 - 9,0) | 99 | 2,4 (1,4 - 4,0) | 100 | 2,2 (1,1 - 4,7) | 99 |
| Anti-inflammatory and antirheumatic products | 1 | 0,5 (0,2 - 1,1) | 51 | 0,6 (0,2 - 1,5) | 52 | 0,2 (0 - 1,2) | 97 | 0,3 (0.1 - 0,9) | 89 |
|  | 2 | 0,3 (0,1 - 0,7) | 28 | 0,4 (0,2 - 0,9) | 35 | 0,2 (0 - 0,6) | 88 | 0,3 (0,1 - 0,7) | 91 |
|  | 3 | 1,1 (0,3 - 3,8) | 98 | 1,6 (0,5 - 5,2) | 99 | 0,7 (0,2 - 2,9) | 100 | 1,8 (0,7 - 4,5) | 100 |
| Antibacterials | 1 | 6,7 (5,0 - 8,9) | 95 | 6,4 (4,4 - 9,2) | 96 | 5,1 (3,2 - 8,1) | 100 | 3,3 (2,5 - 4,5) | 95 |
|  | 2 | 5,1 (3,6 - 7,2) | 96 | 5,2 (3,4 - 8,0) | 97 | 4,3 (3,1 - 6,0) | 99 | 3,1 (2,5 - 3,9) | 94 |
|  | 3 | 6,8 (5,5 - 8,4) | 94 | 7,3 (5,8 - 9,2) | 95 | 5,8 (4,6 - 7,4) | 99 | 4,9 (4,2 - 5,9) | 94 |
| Antihypertensives | 1 | 0,1 (0 - 0,7) | 51 | 0,1 (0 - 1,5) | 53 | 0,1 (0 - 0,8) | 94 | 0,3 (0,1 - 0,8) | 88 |
|  | 2 | 0,3 (0,1 - 0,9) | 70 | 0,5 (0,2 - 1,3) | 74 | 0,2 (0 - 1,0) | 98 | 0,2 (0,1 - 0,7) | 90 |
|  | 3 | 0,4 (0,1 - 1,2) | 89 | 0,5 (0,1 - 1,9) | 91 | 0,4 (0,1 - 1,6) | 97 | 0,5 (0,2 - 1,4) | 98 |
| Antiprotozoals | 1 | 0,2 (0 - 0,5) | 0 | 0,2 (0 - 1,1) | 0 | 0,2 (0,1 - 0,8) | 94 | 0,1 ( 0 - 0,4) | 11 |
|  | 2 | 0,2 (0,1 - 0,3) | 0 | 0,2 (0 - 0,5) | 0 | 0,2 (0,1 - 0,5) | 94 | 0,1 (0,1 - 0,2) | 0 |
|  | 3 | 0,2 (0,1 - 0,6) | 93 | 0,2 (0 - 0,9) | 95 | 0,4 (0,2 - 0,8) | 96 | 0,2 (0,1 - 0,5) | 95 |
| Antithrombotic agents | 1 | 3,1 (1,0 - 8,7) | 99 | 6,6 (2,9 - 14,3) | 99 | 1,7 (0,9 - 2,9) | 99 | 2,5 (1,9 - 3,4) | 88 |
|  | 2 | 3,2 (0,8 - 12,1) | 99 | 8,8 (2,9 - 24,2) | 100 | 1,9 (1,1 - 3,5) | 99 | 3,2 (2,3 - 4,2) | 94 |
|  | 3 | 4,5 (1,1 - 16,5) | 100 | 12,4 (0.4 - 32,5) | 100 | 2,1 (1,2 - 3,6) | 99 | 3,0 (1,8 - 5,0) | 99 |
| Antivirals | 1 | 0,1 (0 - 0,7) | 0 | 0,1 (0 - 1,1) | 0 | 0,3 (0,1 - 0,8) | 96 | 0,1 (0 - 0,5) | 62 |
|  | 2 | 0,3 (0,1 - 0,6) | 47 | 0,2 (0,1 - 0,6) | 51 | 0,1 (0 - 0,7) | 98 | 0 (0 - 0,5) | 55 |
|  | 3 | 0,4 (0,2 - 1,0) | 78 | 0,3 (0,1 - 1,0) | 83 | 0,4 (0,1 - 1,1) | 98 | 0,2 (0 - 0,7) | 92 |
| Corticosteroids | 1 | 0,9 (0,4 - 2,2) | 89 | 1,4 (0,5 - 3,3) | 90 | 0,5 (0,3 - 0,8) | 94 | 0,4 (0,2 - 0,8) | 88 |
|  | 2 | 0,5 (0,2 - 1,3) | 90 | 0,6 (0,2 - 2,0) | 91 | 0,3 (0,2 - 0,5) | 95 | 0,3 (0,2 - 0,6) | 88 |
|  | 3 | 0,7 (0,3 - 1,5) | 89 | 0,9 (0,4 - 2,2) | 91 | 0,3 (0,2 - 0,5) | 98 | 0,3 (0,2 - 0,6) | 95 |
| Cough and cold preparations | 1 | 0,4 (0,2 - 0,9) | 0 | 0,6 (0,4 - 1,0) | 11 | 0 (0 - 0,2) | 0 | 0 (0 - 0,1) | 0 |
|  | 2 | 0,3 (0,1 - 1,2) | 76 | 0,6 (0,2 - 2,0) | 80 | 0 (0 - 0,1) | 42 | 0 (0 - 0,3) | 0 |
|  | 3 | 0,3 (0,1 - 1,1) | 70 | 0,6 (0,2 - 1,9) | 74 | 0 (0 - 0,1) | 33 | 0,1 (0 - 0,1) | 26 |
| Drugs for obstructive airway diseases | 1 | 2,0 (1,0 - 3,8) | 75 | 2,4 (1,3 - 4,6) | 79 | 0,7 (0,2 - 2,9) | 96 | 1,3 (0,9 - 1,8) | 83 |
|  | 2 | 2,8 (1,8 - 4,4) | 80 | 3,4 (2,5 - 4,7) | 86 | 0,7 (0,2 - 2,4) | 96 | 1,2 (0,9 - 1,5) | 71 |
|  | 3 | 1,6 (0,7 - 3,5) | 80 | 2,1 (1,0 - 4,1) | 86 | 0,8 (0,2 - 2,4) | 98 | 1,1 (0,8 - 1,5) | 86 |
| Drugs used in diabetes | 1 | 0,6 (0,3 - 1,2) | 30 | 0,9 (0,5 - 1,5) | 10 | 0,5 (0,1 - 1,7) | 91 | 0,7 (0,5 - 1,2) | 89 |
|  | 2 | 0,8 (0,5 - 1,4) | 66 | 1,1 (0,7 - 1,7) | 70 | 0,7 (0,2 - 2,5) | 97 | 1,1 (0,8 - 1,4) | 82 |
|  | 3 | 1,7 (1,0 - 2,8) | 80 | 1,6 (1,1 - 2,2) | 79 | 0,9 (0,2 - 2,9) | 99 | 1,3 (0,9 - 1,8) | 95 |
| Nasal preparations | 1 | 0,8 (0,4 - 1,8) | 84 | 1,1 (0,6 - 2,3) | 87 | 0,2 (0 - 1,0) | 90 | 0,3 (0,1 - 0,8) | 75 |
|  | 2 | 0,6 (0,2 - 1,6) | 82 | 0,7 (0,3 - 1,9) | 87 | 0,2 (0 - 0,9) | 93 | 0,4 (0,2 - 0,8) | 85 |
|  | 3 | 0,4 (0,2 - 1,0) | 82 | 0,6 (0,3 - 1,3) | 86 | 0,3 (0,1 - 0,9) | 95 | 0,4 (0,2 - 0,7) | 86 |
| Psychoanaleptics | 1 | 2,4 (1,3 - 4,4) | 86 | 2,1 (1,0 - 4,6) | 89 | 3,3 (1,9 - 5,7) | 98 | 2,1 (1,2 - 3,5) | 96 |
|  | 2 | 2,2 (1,2 - 3,7) | 92 | 1,8 (0,9 - 3,6) | 94 | 2,4 (1,3 - 4,4) | 98 | 1,6 (0,8 - 3,1) | 97 |
|  | 3 | 1,4 (0,7 - 2,8) | 94 | 1,6 (0,7 - 3,4) | 96 | 2,3 (1,1 - 4,4) | 99 | 1,4 (0,7 - 3,1) | 99 |
| Psycholeptics | 1 | 0,9 (0,5 - 1,8) | 42 | 1,6 (1,0 - 2,6) | 35 | 0,5 (0,1 - 2,3) | 95 | 0,9 (0,5 - 1,8) | 82 |
|  | 2 | 0,6 (0,3 - 1,2) | 62 | 0,8 (0,4 - 1,6) | 66 | 0,3 (0,1 - 1,3) | 76 | 0,7 (0,4 - 1,1) | 75 |
|  | 3 | 0,6 (0,3 - 1,2) | 81 | 0,9 (0,5 - 1,6) | 85 | 0,5 (0,2 - 1,5) | 91 | 0,8 (0,5 - 1,3) | 93 |

# **Table S9.** Sensitivity analysis: effects of excluding Canadian cohorts on pooled prevalence estimates in non-hospitalized pregnant women with COVID-19 versus non-pregnant women with COVID-19

|  |  | **Non-hospitalized pregnant women**  **with COVID-19** | | | | **Non-pregnant women**  **with COVID-19** | | | |
| --- | --- | --- | --- | --- | --- | --- | --- | --- | --- |
|  | *Trim* | *Original analysis* | *I^2^* | *Sensitivity analysis* | *I^2^* | *Original analysis* | *I^2^* | *Sensitivity analysis* | *I^2^* |
| Analgesics | 1 | 2,8 (1,6 - 4,7) | 78 | 2,6 (1,1 - 5,9) | 85 | 4,1 (2,7 - 6,2) | 99 | 4,3 (2,5 - 7,5) | 95 |
|  | 2 | 1,7 (0,7 - 4,1) | 88 | 2,4 (1,0 - 5,6) | 91 | 3,8 (2,0 - 7,0) | 99 | 3,8 (1,5 - 9,4) | 98 |
|  | 3 | 3,6 (1,7 - 7,7) | 98 | 3,7 (1,1 - 11,4) | 99 | 3,9 (2,2 - 6,9) | 99 | 4,1 (1,8 - 9,0) | 99 |
| Anti-inflammatory and antirheumatic products | 1 | 0,5 (0,2 - 1,3) | 57 | 0,8 (0,4 - 1,6) | 61 | 1,1 (0,2 - 5,5) | 97 | 2,6 (1,5 - 4,6) | 88 |
|  | 2 | 0,4 (0,2 - 0,8) | 41 | 0,5 (0,3 - 0,9) | 52 | 1,2 (0,2 - 5,7) | 97 | 2,7 (1,6 - 4,4) | 96 |
|  | 3 | 1,4 (0,4 - 4,9) | 98 | 2,2 (0,7 - 6,9) | 99 | 1,3 (0,3 - 6,2) | 98 | 3,0 (1,9 - 4,6) | 97 |
| Antibacterials | 1 | 6,9 (5,0 - 9,3) | 94 | 6,6 (4,3 - 9,9) | 96 | 5,8 (3,5 - 9,4) | 99 | 5,3 (2,5 - 10,6) | 99 |
|  | 2 | 5,0 (3,4 - 7,2) | 96 | 5,1 (3,1 - 8,2) | 97 | 6,1 (4,0 - 9,2) | 99 | 5,7 (3,1 - 10,2) | 99 |
|  | 3 | 6,9 (5,4 - 8,7) | 94 | 7,4 (5,7 - 9,6) | 95 | 6,1 (4,0 - 9,3) | 99 | 5,8 (3,2 - 10,3) | 99 |
| Antihypertensives | 1 | 0,1 (0 - 0,8) | 57 | 0,1 (0 - 1,7) | 61 | 0,3 (0 - 1,8) | 98 | 0,4 (0,1 - 1,4) | 95 |
|  | 2 | 0,2 (0,1 - 0,8) | 64 | 0,4 (0,1 - 1,3) | 69 | 0,3 (0,1 - 1,6) | 99 | 0,4 (0,1 - 1,2) | 97 |
|  | 3 | 0,4 (0,1 - 1,4) | 89 | 0,6 (0,1 - 2,5) | 91 | 0,3 (0,1 - 1,6) | 99 | 0,4 (0,1 - 1,2) | 98 |
| Antiprotozoals | 1 | 0,2 (0,1 - 0,6) | 0 | 0,2 (0,1 - 0,8) | 0 | 0,4 (0,2 - 0,6) | 83 | 0,3 (0,1 - 0,6) | 10 |
|  | 2 | 0,2 (0,1 - 0,3) | 0 | 0,2 (0,1 - 0,3) | 0 | 0,3 (0,2 - 0,5) | 87 | 0,2 (0,1 - 0,4) | 69 |
|  | 3 | 0,2 (0,1 - 0,8) | 93 | 0,3 (0,1 - 1,2) | 96 | 0,3 (0,2 - 0,5) | 89 | 0,2 (0,1 - 0,4) | 80 |
| Antithrombotic agents | 1 | 3,3 (1,0 - 10,4) | 99 | 8,4 (3,8 - 17,7)* | 99 | 0,6 (0,3 - 1,3) | 99 | 0,9 (0,3 - 2,5)* | 95 |
|  | 2 | 3,3 (0,7 - 14,7) | 99 | 11,4 (3,6 - 31,0)* | 100 | 0,7 (0,3 - 1,4) | 99 | 1,1 (0,5 - 2,5)* | 96 |
|  | 3 | 5,2 (1,2 - 20,6) | 100 | 17,4 (6,3 - 39,8)* | 100 | 0,7 (0,3 - 1,4) | 99 | 1,1 (0,5 - 2,5)* | 96 |
| Antivirals | 1 | 0,1 (0 - 0,9) | 0 | 0,1 (0 - 2,2) | 0 | 0,2 (0,1 - 0,6) | 94 | 0,2 (0,1 - 0,7) | 87 |
|  | 2 | 0,3 (0,1 - 0,6) | 50 | 0,3 (0,1 - 0,8) | 57 | 0,2 (0,1 - 0,6) | 94 | 0,2 (0,1 - 0,8) | 90 |
|  | 3 | 0,4 (0,2 - 1,1) | 78 | 0,3 (0,1 - 1,2) | 85 | 0,3 (0,1 - 0,6) | 94 | 0,3 (0,1 - 0,7) | 92 |
| Corticosteroids | 1 | 0,8 (0,3 - 2,5) | 87 | 1,4 (0,5 - 4,0) | 89 | 2,1 (1,1 - 4,1) | 100 | 2,7 (1,1 - 6,5) | 98 |
|  | 2 | 0,5 (0,2 - 1,5) | 89 | 0,8 (0,2 - 2,5) | 91 | 2,1 (1,0 - 4,2) | 100 | 2,6 (0,9 - 7,0) | 99 |
|  | 3 | 1,0 (0,5 - 2,1) | 89 | 1,1 (0,5 - 2,6) | 91 | 2,0 (1,0 - 3,8) | 100 | 2,4 (0,9 - 6,0) | 99 |
| Cough and cold preparations | 1 | 0,5 (0,2 - 1,1) | 0 | 0,7 (0,4 - 1,2) | 0 | 0,2 (0 - 2,0) | 96 | 1,4 (0,5 - 4,0) | 87 |
|  | 2 | 0,4 (0,1 - 1,5) | 73 | 0,8 (0,3 - 2,7) | 78 | 0,2 (0 - 1,9) | 97 | 1,2 (0,4 - 3,7) | 89 |
|  | 3 | 0,4 (0,1 - 1,4) | 67 | 0,8 (0,3 - 2,5) | 71 | 0,2 (0,2 - 0,3) | 98 | 1,3 (0,4 - 4,1) | 95 |
| Drugs for obstructive airway diseases | 1 | 1,8 (0,8 - 3,7) | 75 | 2,1 (0,9 - 4,5) | 80 | 1,7 (0,3 - 9,0) | 91 | 3,8 (2,1 - 6,7) | 93 |
|  | 2 | 2,6 (1,6 - 4,2) | 78 | 3,1 (2,2 - 4,5) | 85 | 2,0 (0,4 - 9,8) | 93 | 4,3 (3,1 - 5,9) | 95 |
|  | 3 | 1,4 (0,6 - 3,5) | 80 | 1,9 (0,8 - 4,3) | 87 | 1,8 (0,3 - 9,2) | 97 | 3,9 (2,6 - 5,7) | 98 |
| Drugs used in diabetes | 1 | 0,6 (0,3 - 1,3) | 26 | 0,9 (0,5 - 1,7) | 0 | 0,4 (0,1 - 1,7) | 98 | 0,7 (0,5 - 1,2) | 65 |
|  | 2 | 0,7 (0,4 - 1,3) | 52 | 1,0 (0,7 - 1,6) | 56 | 0,5 (0,1 - 1,9) | 98 | 0,8 (0,5 - 1,2) | 85 |
|  | 3 | 1,6 (0,9 - 3,0) | 82 | 1,5 (1,0 - 2,3) | 83 | 0,4 (0,1 - 1,7) | 98 | 0,6 (0,4 - 1,0) | 92 |
| Nasal preparations | 1 | 0,8 (0,3 - 1,9) | 80 | 1,2 (0,5 - 2,6) | 84 | 0,3 (0 - 2,2) | 97 | 1,4 (0,6 - 3,2) | 94 |
|  | 2 | 0,7 (0,3 - 1,8) | 77 | 0,9 (0,3 - 2,2) | 85 | 0,4 (0,1 - 2,3) | 98 | 1,5 (0,8 - 3,1) | 97 |
|  | 3 | 0,5 (0,2 - 1,1) | 80 | 0,7 (0,3 - 1,4) | 84 | 0,3 (0 - 1,9) | 98 | 1,2 (0,5 - 2,8) | 98 |
| Psychoanaleptics | 1 | 2,1 (1,1 - 3,8) | 81 | 1,7 (0,8 - 3,5) | 86 | 4,7 (3,0 - 7,3) | 99 | 3,4 (2,0 - 5,6) | 98 |
|  | 2 | 1,8 (1,1 - 3,1) | 89 | 1,4 (0,8 - 2,6) | 92 | 4,7 (3,1 - 7,2) | 99 | 3,5 (2,2 - 5,4) | 98 |
|  | 3 | 1,4 (0,8 - 2,5) | 93 | 1,2 (0,6 - 2,6)** | 95 | 4,8 (3,1 - 7,4) | 99 | 3,5 (2,2 - 5,7)** | 99 |
| Psycholeptics | 1 | 0,9 (0,4 - 1,9) | 25 | 1,8 (1,5 - 2,1) | 3 | 1,2 (0,2 - 5,9) | 94 | 2,6 (1,8 - 3,9) | 73 |
|  | 2 | 0,7 (0,3 - 1,3) | 39 | 1,0 (0,6 - 1,6) | 38 | 1,2 (0,2 - 6,2) | 95 | 2,7 (1,5 - 4,7) | 87 |
|  | 3 | 0,7 (0,4 - 1,3) | 76 | 1,0 (0,6 - 1,7)* | 82 | 1,3 (0,3 - 6,2) | 95 | 2,7 (1,8 - 4,1)* | 93 |

* The pooled prevalence confidence intervals did overlap in the original analysis but did not overlap after excluding Canadian cohorts.
** The pooled prevalence confidence intervals did not overlap in the original analysis but did overlap after excluding Canadian cohorts.
